# Predicting mortality dynamics in cancer patients: A machine learning approach to pre-death events

**DOI:** 10.1371/journal.pone.0331650

**Published:** 2025-09-09

**Authors:** Tatsuki Yamamoto, Minoru Sakuragi, Yuzuha Tuji, Yuji Okamoto, Eiichiro Uchino, Motoko Yanagita, Manabu Muto, Mayumi Kamada, Yasushi Okuno

**Affiliations:** 1 Department of Biomedical Data Intelligence, Graduate School of Medicine, Kyoto University, Kyoto, Japan; 2 Department of Nephrology, Graduate School of Medicine, Kyoto University, Kyoto, Japan; 3 Institute for the Advanced Study of Human Biology (ASHBi), Kyoto University, Kyoto, Japan; 4 Department of Clinical Oncology, Graduate School of Medicine, Kyoto University, Kyoto, Japan; 5 Department of Data Science, School of Frontier Engineering, Kitasato University, Kanagawa, Japan; Chinese Academy of Sciences, CHINA

## Abstract

Capturing the dynamic changes in patients’ internal states as they approach death due to fatal diseases remains a major challenge in understanding individual pathologies and improving end-of-life care. However, existing methods primarily focus on specific test values or organ dysfunction markers, failing to provide a comprehensive view of the evolving internal state preceding death. To address this, we analyzed electronic health record (EHR) data from a single institution, including 8,976 cancer patients and 77 laboratory parameters, by constructing continuous mortality prediction models based on gradient-boosting decision trees and leveraging them for temporal analyses. We applied Shapley Additive exPlanations (SHAP) to assess the contribution of individual features over time and employed a SHAP-based clustering approach to classify patients into distinct subtypes based on mortality-related feature dynamics. Our analysis identified three distinct clinical patterns in patients near death, with key laboratory parameters—including albumin, C-reactive protein, blood urea nitrogen, and lactate dehydrogenase—playing a critical role. Dimensionality reduction techniques demonstrated that SHAP-based patient stratification effectively captured hidden variations in terminal disease progression, whereas traditional stratification using raw laboratory values failed to do so. These findings suggest that machine learning-driven temporal analysis can reveal clinically meaningful state transitions that conventional approaches overlook, offering new insights into the heterogeneous nature of terminal disease progression. This framework has the potential to enhance personalized risk stratification and optimize individualized end-of-life care strategies by identifying distinct patient trajectories that may inform more targeted interventions.

## Introduction

Elucidating the physiological changes that occur near the end of life is a significant challenge in terminal care. Death arises from temporal changes within a complex internal state involving multiple organs and molecular networks, and this process exhibits considerable interindividual variability [1]. In many fatal diseases, understanding the near-death internal state dynamics and the associated variations between patients is crucial to elucidate disease pathophysiology and enhance the personalization of end-of-life care [2,3]. The changes in organ function and prognostic factors based on the patterns of laboratory test values and vital signs before death have been previously investigated [4–8]. However, the focus has been mostly on the statistical analysis of specific laboratory test values or the progression of organ dysfunction markers. Consequently, these studies did not directly capture the dynamics across the entire internal state leading to death.

Cancer, a representative fatal disease, exhibits changes in symptoms and test values before death, with considerable variation throughout its progression [9–14]. Analysis of the patterns of internal state changes in patients with terminal cancer could benefit from the integration of machine learning approaches with traditional statistical methods [15] by facilitating refined patient stratification. However, the use of feature–value–based stratification in many studies overlooks the predictive importance of each feature and limits their effectiveness in capturing the dynamics and variations of internal states before death [16,17].

Herein, we propose a novel framework that uses machine-learning-based mortality prediction models and interpretation techniques to estimate temporal changes in key factors associated with mortality. We aimed to use this framework to extract changes in factors that considerably influence mortality and elucidate the dynamics and variations in the internal states of patients with cancer leading to death by exploiting SHapley Additive exPlanations (SHAP) [18] as the model interpretation method. SHAP values quantify the contribution of each feature to the predicted outcome. Stratifying patients based on these values [19–23] enables the identification of key factors associated with outcomes that are often difficult to capture using conventional classifications based on raw feature values [24]. Hence, we extended this method to analyze continuous changes and estimate “SHAP behaviors” that represent the temporal changes in key mortality-related features. This allowed us to characterize the temporal changes in the internal states of each patient with cancer.

In this analysis, we constructed continuous mortality prediction models for patients with cancer using time-series laboratory test data. Second, we compared the SHAP behavior of each laboratory parameter across the entire cohort and identified the key parameters determining patient states. Third, we stratified patients with cancer based on their SHAP values immediately before death and evaluated the SHAP behaviors of the key parameters and the clinical characteristics of each subtype. The findings of this study contribute to elucidating the pathophysiological mechanisms leading to death from fatal diseases, including cancer, and to optimizing personalized care in end-of-life medicine.

## Materials and methods

### Dataset

The data selection process is illustrated in Fig S1-1 in S1 Text. We used the electronic health record (EHR) data from January 2006 to December 2019 available at Kyoto University Hospital to identify patients with cancer who were confirmed dead (n = 9,614). We focused on blood and urine laboratory tests and selected 1,400 laboratory parameters because 97.7% of the test samples in the cancer patient dataset were from blood tests or urinalysis. Subsequently, we extracted the data of patients whose test results for the last one year before their death were available (n = 8,991). Laboratory parameters examined in more than half of the patients were retained, while patients with missing values for these laboratory parameters were excluded. The final dataset comprised data from 8,976 patients with the results of 77 laboratory parameters (Table S2-1 in S1 Text). For multiple tests conducted on the same day, only the result from the first test was used.

Each laboratory test dataset was time-stamped and resampled for each patient using a moving average, with the window width set to 5 days based on the median time between the tests for each patient (Fig S1-2A in S1 Text). Some patients lacked data at certain time points because they had not been tested. Time-series EHR data were labeled to develop the prediction model (Fig S1-2B in S1 Text). For the training of each model predicting death “n” days later, laboratory test data from “n” days before death were labeled as positive, and data from 168 days (24 weeks) before death were labeled as negative. Only patients with data available at both time points were included, ensuring a balanced label ratio. The number of patients differed across datasets from different time points. Based on the data availability, we selected 24 weeks before death as the negative time point. Although selecting a time point distant from death is crucial and helps ensure distinct patient states, data availability decreases as the time point gets further from death. Thus, owing to the trade-off between the distance from death and the amount of data available at each time point, the negative time point was selected as approximately six months before death.

### Continuous mortality prediction model

Independent LightGBM-based [25] models were constructed to predict mortality at each time point from one day to 90 days before death using labeled laboratory parameter datasets (Fig S1-2B in S1 Text). The number of patient samples in the dataset used to construct the model at each time point is shown in Fig S1-3A in S1 Text. For each model, the dataset was split into 80% training and 20% test sets. The training set was further divided in a 4:1 ratio to generate a validation set, which was used for hyperparameter tuning. Hyperparameter optimization for all models was performed using Optuna’s LightGBMTunerCV [26]. The optimal hyperparameters identified for each model were then used for performance evaluation and downstream analyses. We evaluated the model performance using five-fold cross-validation on a per-patient basis using the area under the receiver operating characteristic curve (AUROC) as the evaluation metric (Fig S1-3B in S1 Text). We did not employ additional data balancing techniques, as the class distributions of mortality and survival cases were not severely imbalanced at most time points, as indicated by the confusion matrices (Table S3-1 in S1 Text).

### Estimation of SHAP behaviors and identification of key features

SHAP values were used to quantify and interpret the temporal transition of feature importance for mortality prediction. SHAP is a widely adopted method for explaining machine learning model predictions. The SHAP values represent the contribution of each feature to the model prediction. In a mortality prediction model, a positive SHAP value indicates an increased risk of death, whereas a negative value indicates a decreased risk. In the proposed continuous mortality prediction models, the SHAP values of each feature were calculated for each patient at each time point. The importance of the features was then compared from the distribution of SHAP values by calculating the mean SHAP value of each feature at each time point across patients. We applied the SHAP framework independently to each prediction model constructed at different time points to obtain feature importance values specific to each time point. The temporal changes in these mean SHAP values of each feature were referred to as “SHAP behaviors” and were analyzed for a systematic comparison of the importance of features at different time points. To enable consistent comparison of feature importance across different time points, we scaled the SHAP values at each time point by dividing them by the maximum SHAP value from the corresponding prediction model. This normalization allowed us to visualize the relative importance of each feature over time. In the corresponding figures, the vertical axis represents the mean of the scaled SHAP values at each time point. These normalized SHAP behaviors enabled us to track the dynamic contribution of each feature to mortality risk over time.

First, we estimated the SHAP behavior of each laboratory parameter for each patient in the test dataset using continuous mortality prediction models. The mean of the SHAP values of each parameter was then visualized. In addition, we focused on the parameters that had a particularly strong influence on mortality prediction. How SHAP behaviors reflect patient state dynamics was assessed based on clinical knowledge by comparing the distribution of SHAP behaviors with the distribution of the actual laboratory test results. To identify the factors influencing the mortality of a patient, the SHAP value analysis utilized only the data labeled as positive and correctly predicted the same.

### Visualization of the patient state dynamics

We visualized the SHAP behaviors preceding death using dimensionality reduction. UMAP [27], t-SNE [28], and principal component analysis (PCA) were used for dimensionality reduction, which was performed separately for the laboratory parameters and SHAP values. The hyperparameters for UMAP were set as follows: metric, euclidean; n_neighbors, 10; min_dist, 0.5; and random_seed, 0. The settings were determined considering the density and separation of the distributions (Fig S1-4 in S1 Text). Dimensionality reduction was performed using the determined parameters, and the results were plotted in two dimensions to visualize the transition of the data points over time.

### Subtype analysis of patient state transition

To analyze the transition patterns leading to the near-death state, we performed hierarchical clustering using the SHAP values from one day before death as they showed the most extensive spread in the UMAP plot. The Ward method with the Euclidean distance was employed, and a threshold was set to identify patient state subtypes with different distribution patterns in the UMAP plot. For comparison, we performed hierarchical clustering of the laboratory parameter data using the same method, and the differences in distribution between the subtypes derived based on the SHAP values and those derived based on the laboratory data were compared.

Subsequently, we examined the differences in SHAP behaviors among subtypes. For each subtype, the distribution of the SHAP values of all the selected laboratory parameters was estimated from one day to 90 days before death. To investigate how differences in SHAP behaviors reflect actual changes in patient states, differences in the parameter data, sex, age group, and the distribution of cancer types within each subtype were statistically analyzed. Further, the clinical characteristics of each subtype were evaluated.

### Ethical statement and informed consent

The dataset was generated and reviewed using the clinical information obtained from the EMR of our institution. This study was conducted using data collected exclusively during routine medical practice, in accordance with the principles of the Declaration of Helsinki. In compliance with Japanese laws and regulations, informed consent was obtained through an opt-out approach. All study details and consent procedures were provided in written form, ensuring that participants received comprehensive information and that their decisions were properly documented. This approach was approved by the Ethical Review Board of Kyoto University as an appropriate method of consent for this type of research. We ensured ethical compliance by publicly providing detailed information about the study, including its purpose, the nature of the data used, and the rights of participants to withdraw, on the Kyoto University Hospital website (https://www.kuhp.kyoto-u.ac.jp/outline/research-disclosure.html). The option for participation was clearly presented and easily accessible, ensuring the preservation of participant autonomy. The Ethical Review Board of Kyoto University approved the study (Approval Number R1498), deeming it appropriate for this retrospective analysis. Access to the dataset was initiated on March 1, 2022, and completed on March 31, 2022. Data analysis for this study was subsequently conducted through August 2022. During data collection, the authors did not access any personally identifiable information of the participants.

### Computational environments

All analyses, including statistical tests, were conducted in the python environment. The required versions are as follows: Python (v3.9), LightGBM (v3.0.0), shap (v0.46.0), and optuna (v2.0.0). A complete list of dependencies and implementation details is available in the code repository: https://github.com/clinfo/SHAP_behavior_estimate.git.

## Results

### Mortality prediction models

We utilized EHR data from Kyoto University Hospital. The dataset included patients with cancer who had died. Data were extracted according to the data selection criteria (See Materials and Methods). Table 1 shows the composition of sex, age groups, and cancer types based on ICD-10 codes (International Statistical Classification of Diseases and Related Health Problems). The final dataset comprised 8,976 patients and 77 laboratory parameters (Table S2-1 in S1 Text).

**Table 1:** Descriptive characteristics of patients whose data were included in the study.

Cancer types	Lip, oral cavity, and pharyngeal cancer	Cancer related to digestive organs	Cancer related to respiratory and intrathoracic organs	Bone and articular cartilage cancer	Melanoma and skin cancer	Mesothelial and soft tissue cancer	Breast cancer	Cancer related to female genital organs	Cancer related to male genital organs	Cancer related to urinary tract	Cancer related to eye, brain, and parts of CNS	Cancer related to thyroid and other endocrine glands	Cancer at secondary and unspecified sites	Lymphoid, hematopoietic, and related cancers	In situ neoplasms	Benign neoplasms	Uncertain or unknown cancer types	All cancer types
**Patients [n]**	206	2944	1055	29	93	156	202	320	261	281	202	76	2052	735	7	132	225	8976
**Age [n(%)]**																		
**20–39 years**	1 (0.5)	51 (1.7)	3 (0.3)	6 (20.7)	6 (6.5)	11 (7.1)	7 (3.5)	16 (5.0)	1 (0.4)	2 (0.7)	38 (18.8)	2 (2.6)	60 (2.9)	48 (6.5)	1 (14.3)	7 (5.3)	18 (8.0)	278 (3.1)
**40–59 years**	34 (16.5)	431 (14.6)	97 (9.2)	9 (31.0)	18 (19.4)	35 (22.4)	79 (39.1)	112 (35.0)	4 (1.5)	23 (8.2)	56 (27.7)	12 (15.8)	461 (22.5)	166 (22.6)	2 (28.6)	21 (15.9)	37 (16.4)	1597 (17.8)
**60–79 years**	121 (58.7)	1946 (66.1)	721 (68.3)	14 (48.3)	47 (50.5)	88 (56.4)	84 (41.6)	155 (48.4)	132 (50.6)	149 (53.0)	91 (45.0)	43 (56.6)	1303 (63.5)	413 (56.2)	2 (28.6)	59 (44.7)	108 (48.0)	5476 (61.0)
**>80 years**	50 (24.3)	516 (17.5)	234 (22.2)	0 (0.0)	22 (23.7)	22 (14.1)	32 (15.8)	37 (11.6)	124 (47.5)	107 (38.1)	17 (8.4)	19 (25.0)	228 (11.1)	108 (14.7)	2 (28.6)	45 (34.1)	62 (27.6)	1625 (18.1)
**Sex [n]**																		
**Male/ Female**	141/ 65	2028/ 916	796/ 259	15/ 14	52/ 41	99/ 57	4/ 198	0/ 320	261/ 0	215/ 66	124/ 78	26/ 50	1191/ 861	417/ 318	2/ 5	67/ 65	128/ 97	5566/ 3410

The cancer types were categorized using ICD10 codes, with “CNS” in the table representing “Central nervous system.”

**Fig 1:**
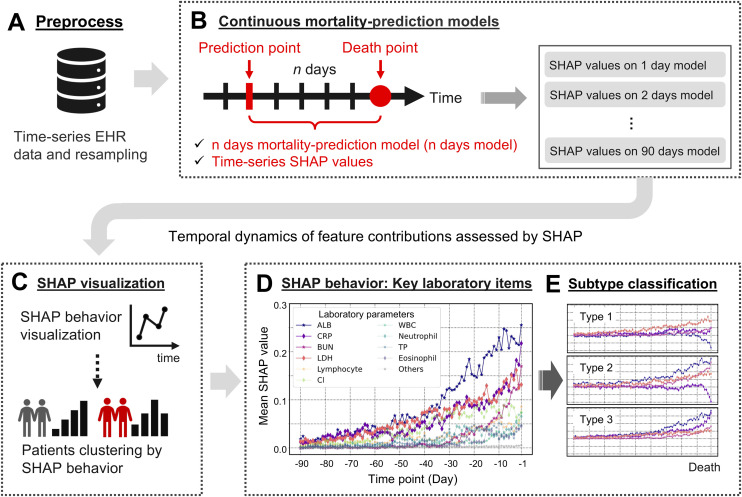
Overview of the analysis. **(A)** The original electronic health record (EHR) data were resampled to generate time-series EHR data. **(B)** At each time point from one day to 90 days before death, machine learning-based mortality prediction models were constructed. SHapley Additive exPlanations (SHAP) were then applied to each mortality prediction model to calculate SHAP values sequentially. **(C)** The calculated SHAP values were aggregated by feature, and the temporal changes, referred to as “SHAP behaviors,” were estimated. **(D)** The “SHAP behaviors” were visualized, and the key features in patient states were extracted and interpreted based on clinical insights. **(E)** Patients were stratified based on the patterns of SHAP values immediately before death. SHAP behaviors of key features in each patient group were estimated to identify the subtypes of changes in patient states leading to death, and the clinical backgrounds of patients within each subtype were evaluated. Abbreviations: ALB, serum albumin; CRP, C-reactive protein; BUN, blood urea nitrogen; LDH, lactate dehydrogenase; Cl-, chloride; WBC, white blood cell count; TP, total protein.

The dataset was resampled using moving averages to generate time-series laboratory test data (Fig 1A). Continuous mortality prediction models were constructed using laboratory test data from “n” days before death at specific time points to predict mortality “n” days later. Models were built for each time point from one day to 90 days before death (See Methods). The mean AUROC values after five-fold cross-validation were 0.965 (± 0.008) for one day before death, 0.851 (± 0.019) for 30 days before death, 0.721 (± 0.011) for 60 days before death, and 0.625 (± 0.019) for 90 days before death. Models closer to the time of death demonstrated better performance. The performance of the predictive models at each time point and the confusion matrices are provided in S3 File in S1 Text.

### SHAP behavior analysis

**Fig 2:**
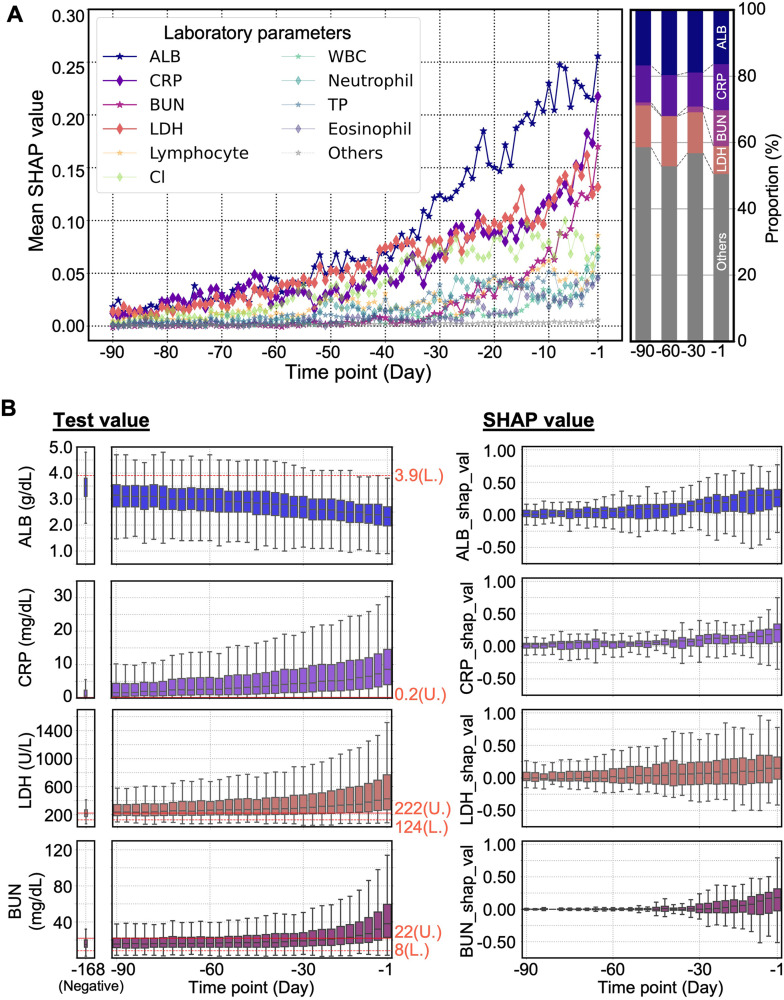
SHAP behaviors and SHAP value distribution of each laboratory parameter. **(A)** (Left) SHAP behaviors of the top 10 influential parameters. The vertical axis shows the mean of the scaled SHAP values divided by the maximum value of SHAP. “Others” represents the remaining 67 parameters, excluding the top 10 influential parameters. The plot represents discrete daily transitions. The larger the mean value at each time point, the greater the importance of the corresponding parameter on the patient’s state. (Right) Transition of the proportion of mean SHAP values for ALB, CRP, BUN, LDH, and other parameters. The proportion was calculated as the percentage of the absolute mean SHAP value of each parameter relative to its sum. **(B)** The transition of (left) laboratory test values and (right) SHAP values of ALB, CRP, LDH, and BUN. Both were aggregated every 3 days up to 90 time points. The dashed line represents the upper limit (U.), and the dotted line represents the lower limit (L.) of the reference values. The SHAP values were scaled based on the maximum value.

We applied SHAP to determine the importance of each feature for mortality prediction. The mortality prediction models calculated SHAP values from the feature values at each time point. We calculated the temporal changes in mean SHAP values of each feature, referred to as “SHAP behaviors” (please refer to the “Estimation of SHAP behaviors and identification of key features” subsection in the Materials and methods section). The top 10 features with the highest mean SHAP values one day before death were serum albumin (ALB), C-reactive protein (CRP), blood urea nitrogen (BUN), lactate dehydrogenase (LDH), lymphocyte count, chloride (Cl-), white blood cell count (WBC), neutrophil count, total protein (TP), and eosinophil count. These 10 features were identified as key contributors to the patient state preceding death. These parameters exhibited temporal changes in their values and rankings leading to death, as shown by their SHAP behaviors over the 90 days preceding death, thus indicating their importance (Fig 2A).

ALB, CRP, and LDH were consistently ranked among the top three influential features at most time points. The importance of these features varied over time, as shown in the bar graph in Fig 2A. 90 days before death, ALB, CRP, and LDH levels were comparable. However, 60 and 30 days before death, ALB exhibited the highest importance, whereas the relative importance of CRP and LDH levels decreased. In contrast, BUN exhibited a distinctive pattern. BUN ranked low until approximately 30 days before death, when its importance sharply increased to become the third most influential factor leading to death, following ALB and CRP. For reference, S4 File in S1 Text provides the mean SHAP values of all 77 features, including those outside the top 10 influential laboratory parameters, one day before death.

We plotted the transition of SHAP values and laboratory test values for ALB, CRP, LDH, and BUN, which were highly influential particularly in mortality prediction and crucial for understanding the patient state changes (Fig 2B). ALB values gradually decreased as the patient approached death, and the corresponding SHAP values tended to increase. Thus, decreasing ALB levels contributed to driving the patient toward mortality. Conversely, CRP, LDH, and BUN values gradually increased over time, and the corresponding SHAP values increased. Thus, the increasing values of these tests were associated with the patient state, indicating impending death. For reference, S5 File in S1 Text provides laboratory reference values for ALB, CRP, BUN, and LDH.

### Time-series SHAP value trajectories

Dimensionality reduction was performed to capture the spatial transition of the patient’s internal state leading to death. Because performing dimensionality reduction of data containing missing values is challenging, we used only those features with less than 10% missing values at all 90 time points; thus, 29 features were considered (Table S6-1 in S1 Text). Patient samples with missing values for the selected features were excluded.

**Fig 3:**
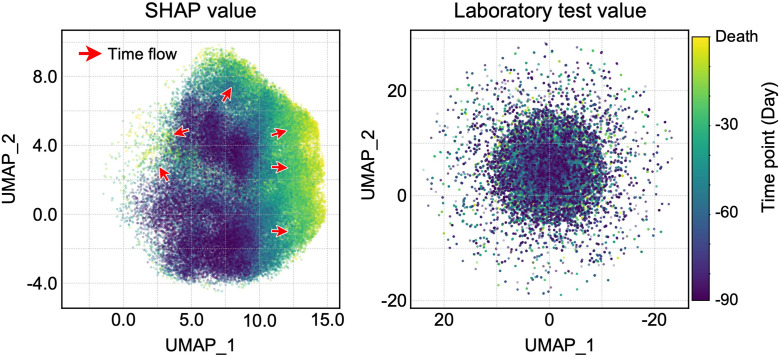
UMAP plots of the patient state trajectory. Two-dimensional UMAP plots generated using SHAP values (left) and standeardrized laboratory test values (right). The horizontal and vertical axes represent the first and second UMAP components, respectively. For laboratory test values, each feature was standardized before dimensionality reduction. The color gradients indicates the time points, with the color changing from navy to yellow as time progressed. As indicated by the red arrows, temporal transitions in the distribution of patient states are observed only in the SHAP-based plot. Only data points with positive labels (i.e., correctly predicted death) are shown in the plot.

The SHAP trajectories visualized using uniform manifold approximation and projection (UMAP) are shown in Fig 3. When laboratory test values were used for analysis, no discernible changes were observed in the transition of the patient states. In contrast, employing SHAP values indicated a temporal transition in the distribution representing SHAP behaviors. Although similar trends were observed during t-distributed stochastic neighbor embedding (t-SNE) and PCA, temporal transitions in the distribution were most effectively depicted in the UMAP (Fig 3 and Fig S6-1A in S1 Text). These findings demonstrate that time-series SHAP values can be used to visualize trajectories potentially culminating in death. Additionally, the distribution of the laboratory test values of ALB, CRP, LDH, and BUN could be visualized (Fig S6-1B in S1 Text) using the UMAP plot (Fig 3).

### Patient states stratified based on SHAP trends

**Fig 4:**
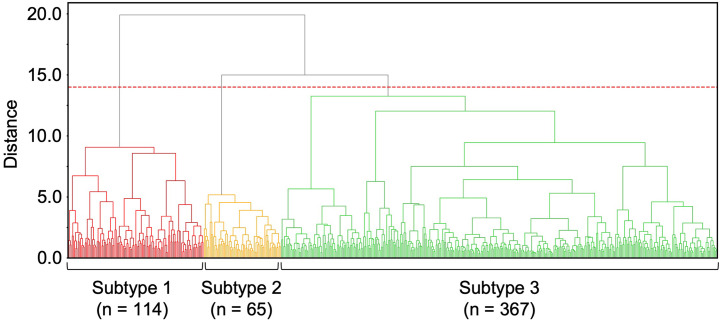
Classification of patient state subtypes based on SHAP values. Classification of patient state subtypes one day before death. The horizontal axis represents the individual patients. Clustering was performed using Ward’s method and the Euclidean distance. The dashed red line indicates the threshold value. The number of clusters was determined by considering the results of the ELBOW and silhouette methods (S7 File in S1 Text). The number of patient samples for each subtype is shown at the bottom of the figure.

SHAP behaviors of laboratory parameters were more dispersed as death approached (Fig 3), indicating the existence of patient state subtypes immediately before death. Hence, we performed hierarchical clustering using SHAP values from one day before death to stratify patient states immediately before death. Three patient state subtypes were identified (Fig 4 and S7 File in S1 Text). In contrast, stratification using raw laboratory values did not effectively capture the transition differences between the subtypes (S8 File in S1 Text).

### SHAP behaviors across different subtypes in ALB, CRP, LDH, and BUN values

**Fig 5:**
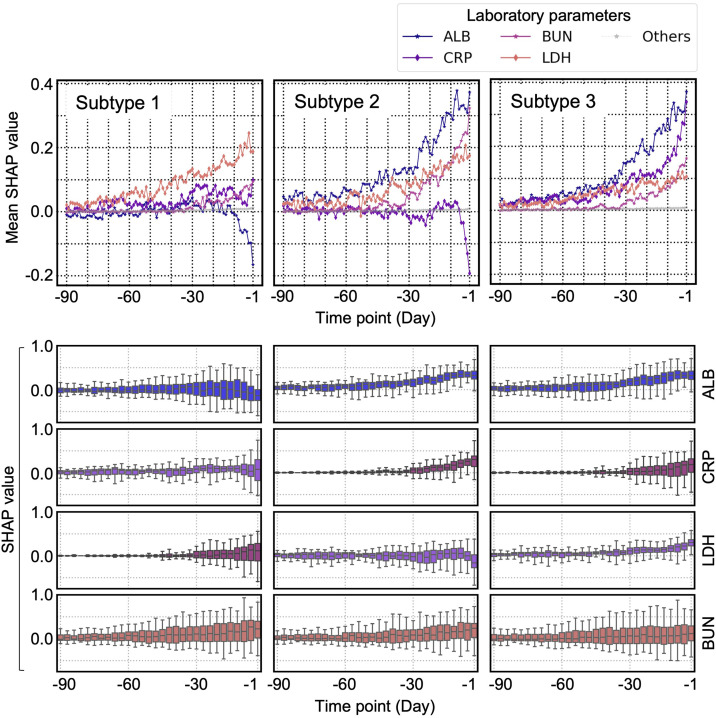
The SHAP behaviors of ALB, CRP, BUN, and LDH in each subtype. (Top) SHAP behaviors of ALB, CRP, LDH, BUN, and other laboratory parameters in each trajectory lead to specific patient state subtypes. “Others’‘ represent the remaining 73 items. The plot represents discrete transitions daily. (Bottom) Distribution of SHAP values of each laboratory parameter, aggregated across 90 time points at 3-day intervals.

In the context of patients’ internal states subtypes, the term “trajectory” refers to the pattern of SHAP behavior over the 90 days preceding death for each subtype. For example, the trajectory of subtype 1 refers to a 90-day process finally leading to a patient state defined as “subtype 1.” Among the top 10 influential parameters, ALB, CRP, LDH, and BUN exhibited particularly high SHAP values and distinct SHAP behaviors across different subtypes (Fig 5). The transitions of the top 10 influential parameters are shown in S9 File in S1 Text. In addition, Table 2 shows the subtype-specific mean and median laboratory test values of ALB, CRP, BUN, and LDH one day before death (Figs 4 and 5).

**Table 2:** Laboratory test values immediately before death in each subtype.

	Overall	Subtype 1	Subtype 2	Subtype 3
**Patients [n]**	546	114	65	367
**ALB [g/dL, median (IQR)]**	2.3 (2.0–2.7)	3.1 (2.9–3.3)	2.2 (1.9–2.4)	2.2 (1.9–2.4)
**CRP [mg/dL, median (IQR)]**	8.7 (4.3–14.7)	4.3 (1.6–9.6)	1.9 (1.2–3.0)	11.6 (7.3–17.1)
**BUN [mg/dL, median (IQR)]**	41.3 (25.0-64.6)	29.0 (19.0-52.0)	54.0 (38.0-88.5)	42.0 (25.1-65.2)
**LDH [U/L, median (IQR)]**	428.3 (271.4-771.1)	466.3 (309.5-811.1)	509.0 (340.0-820.3)	406.6 (254.3-738.5)

The median and interquartile range (IQR) of selected parameters were calculated for the entire dataset and for each patient state subtype. Statistical hypothesis testing was performed to identify the differences in the mean values of these parameters across subtypes. Subtype 1 had a significantly higher ALB value than the other subtypes. Subtype 2 had a significantly lower value than the other subtypes, whereas subtype 3 had a significantly higher value. Subtype 1 had significantly lower BUN value than the other subtypes, whereas subtype 2 had the highest BUN value. All tests were performed at a significance level of 1%, with corrections applied for multiple comparisons. Detailed information on statistical hypothesis testing is provided in S10 File in S1 Text. ALB, serum albumin; CRP, C-reactive protein; BUN, blood urea nitrogen; LDH, lactate dehydrogenase.

The results shown in Fig 5 and Table 2 were interpreted based on two clinicians’ findings. In subtype 1, ALB values were maintained until just before death (Table 2), while the SHAP values were highest for LDH over the 90 days preceding death (Fig 5). The trajectory of subtype 1 suggested severe tissue damage or tumor cell infiltration and necrosis. In subtype 2, the SHAP importance of ALB was greatest over the 90 days preceding death, and the involvement of inflammatory markers, such as CRP, remained minimal, indicating that the trajectory of subtype 2 represents severe malnutrition or hepatic dysfunction owing to cancer progression. In subtype 3, the ALB test value was low, the CRP test value was high (Table 2), and the SHAP values of ALB and CRP before death were higher than those in the other trajectories (Fig 5). These characteristics are consistent with clinical features of cachexia observed in patients with terminal cancer, suggesting that the trajectory of subtype 3 represents severe cancer cachexia.

The identified subtypes exhibited clinically interpretable characteristics. During the SHAP analysis, ALB, CRP, LDH, and BUN levels were identified as particularly important. Declining ALB levels are an independent prognostic factor in cancer [29]. Other studies involving patients with cancer have shown increased CRP and LDH levels before death and highlighted their utility as prognostic indicators [30,31]. High BUN levels are also associated with reduced survival rates [32]. Thus, the interpretation of changes in patient status based on SHAP values aligns with the findings from previous studies and highlights the association of low ALB and elevated LDH and CRP levels with inflammation and tissue damage in patients with terminal cancer. Furthermore, in our study, the characteristics of the subtypes based on SHAP behaviors just before death reflected known clinical features of terminal cancer, such as inflammation, malnutrition, and cachexia [33,34].

We explored the differences in sex, age group, and cancer classification based on ICD-10 codes among the subtypes. Subtype 1 exhibited a significantly lower proportion of patients aged 60–79 years (P < 0.01) than the other subtypes (S10 File in S1 Text). Other attributes, such as age, sex, and cancer classification, did not differ significantly between the subtypes. The results of detailed analyses of each subtype are provided in S11 File in S1 Text.

## Discussion

In the present study, we combined machine learning-based mortality prediction models with the SHAP framework to identify key features associated with cancer-related mortality and the temporal changes in patients’ internal states. By examining the SHAP behaviors of influential parameters, we stratified patient states immediately before death based on the contributions of the top 10 influential laboratory parameters, including ALB, CRP, LDH, and BUN. In the current study, SHAP-based clustering effectively captured the clinically relevant transitions in patient states before death, providing novel insights that have been overlooked by studies using conventional analytical methods.

Numerous factors determine the internal states of patients with cancer preceding death; however, individual differences and temporal variations make quantitative evaluation and classification challenging. Conventional analysis methods focusing on prognostic factors and laboratory test value variations have been unable to capture such complex dynamics. In the present study, laboratory test data alone did not yield classifications that effectively reflected differences in internal state trends (S8 File in S1 Text). However, continuous analysis of SHAP behaviors using machine learning-based prediction models revealed subtypes reflecting differences in temporal trends in patient internal states. Thus, machine learning prediction models capture and learn internal state changes that are not apparent in traditional test value analyses. Because machine learning quantifies mortality-related features from vast clinical data, such as SHAP values; these analyses can be more profound than relying on laboratory test data alone.

The clinical significance of our approach lies in its ability to visualize and stratify dynamic internal changes in patients with complex conditions using interpretable machine learning prediction models. In other words, it enables the extraction of early indicators that most strongly influence predicted outcomes from vast learned patterns in machine learning models— an achievement that had been difficult with conventional methods.

Patients with advanced cancer approaching death often experience internal state transitions driven by various factors and pathways. However, early detection of such changes has typically relied on a limited set of subjective observations, objective findings, and basic laboratory values. These conventional indicators alone are insufficient to capture the early, diverse, and dynamic changes in patient states. Our approach makes it possible to extract and stratify patterns of internal state transitions in patients with terminal cancer, even from limited laboratory test information. If such SHAP-based subtypes can be identified early, they may facilitate improved personalization and optimization of end-of-life care, including timely palliative care interventions, advance life planning, and treatment strategies that avoid unnecessary medical interventions and reduce patient burden.

Furthermore, combining time-series analysis and clustering of SHAP values enhances the interpretability of machine learning models, as demonstrated in this study. Although previous studies have shown that patient clustering based on SHAP can capture crucial clinical backgrounds [21,24], the dynamic analysis of SHAP behaviors and identification of patient state subtypes discussed in this study are novel to our knowledge. Incorporating more multimodal data for learning and reasoning could facilitate a more comprehensive understanding of the dynamic patient states preceding death and the underlying mechanisms. Furthermore, the analysis framework from this study could be applied to a broader range of time-varying clinical outcomes beyond cancer-related mortality.

In this study, we conducted a retrospective analysis using electronic medical record data. However, because our approach uses pre-trained models and clustering based on SHAP patterns, it is also applicable to new patients, enabling prospective clustering in real-time clinical practice. Furthermore, our team is currently conducting prospective clinical validations using interpretable artificial intelligence models to support bedside decision making. Therefore, this study holds clinical value both as a pilot investigation demonstrating the feasibility of implementing interpretable artificial intelligence in real-world clinical decision-making and as a novel contribution that advances our understanding of the dynamic internal states of patients with terminal cancer through data-driven stratification.

### Strengths and limitations of the study

The present study had some limitations. First, as this was a retrospective analysis using single-center data, external prospective validations are required to verify the clinical utility of our framework. Second, our analysis used only electronic medical record data obtained during the usual course of practice; hence, we need to consider biases in patient background and time-series data frequency when interpreting the results. Third, direct comparison of SHAP values across models with differing prediction accuracies is difficult when interpreting temporal changes in SHAP values. We focused on trends in relative SHAP importance rather than SHAP values themselves. Future work should explore prediction models that can directly analyze continuous SHAP values. Fourth, owing to the potentially missing and highly variable nature of the dataset, direct analysis of the subtypes of dynamic changes can be challenging, and we could not cluster the entire SHAP value trends as sequential data. More homogeneous data are required for further dynamic cluster analysis. Fifth, although the subtypes based on SHAP values can be clinically reasonable, few apparent differences were observed in age, sex, or disease codes. This indicates that these metrics alone do not capture subtype distinctions. Our current study was limited to routinely available laboratory test values, and we acknowledge that incorporating additional variables such as vital signs, medications, and comorbidities would enhance both the interpretability and robustness of clustering results. However, owing to the nature of terminal cancer care and associated limitations in data availability, especially in advanced or terminal stages, we deliberately focused on laboratory data that were consistently obtainable across the cohort. Finally, although we focused our analysis on 90 days before death in this study, a longer analysis period may reveal mortality-related factors over extended time frames.

This study is one among the first to systematically capture temporal transitions in patient states before death by leveraging SHAP analysis of continuously trained mortality prediction models. In the present study, we elucidated the patient state dynamics leading to death by capturing SHAP behaviors using continuous mortality prediction models for patients with cancer. Our study is valuable in demonstrating, for the first time, the potential of identifying laboratory parameters that are highly influential in patient states, estimating the temporal changes in their importance to patients’ internal states over time, and clustering complex internal states preceding death based on SHAP values. The findings could facilitate the understanding of the pathological mechanisms leading to death from fatal diseases, including cancer, and to enhance personalized end-of-life care.

## Supporting information

S1 Text **S1 File.** Supplemental information of methodology. **S2 File**. Laboratory parameter list. **S3 File**. Performances and confusion matrices of continuous mortality prediction models. **S4 File**. Mean SHAP values of all parameters immediately before death. **S5 File**. Reference values of ALB, CRP, BUN, and LDH. **S6 File**. Details of visualizing changes in patient states using time-series SHAP values. **S7 File**. Evaluation of the number of clusters in patient stratification using SHAP values. **S8 File**. Stratification of patient states using laboratory values. **S9 File**. SHAP behaviors of the top influential items for each subtype. **S10 File**. Statistical tests on laboratory test values, biological sex, age, and cancer type. **S11 File**. Detailed analysis and discussion of the background of the patient state change subtypes.
(ZIP)

## References

[1] Kitano H. Systems biology: a brief overview. Science. 2002;295(5560):1662–4. doi: 10.1126/science.1069492 11872829

[2] Lunney JR, Lynn J, Foley DJ, Lipson S, Guralnik JM. Patterns of functional decline at the end of life. JAMA. 2003;289(18):2387–92. doi: 10.1001/jama.289.18.2387 12746362

[3] Cohen-Mansfield J, Skornick-Bouchbinder M, Brill S. Trajectories of End of Life: A Systematic Review. J Gerontol B Psychol Sci Soc Sci. 2018;73(4):564–72. doi: 10.1093/geronb/gbx093 28977651

[4] Xie Y, Bowe B, Xian H, Balasubramanian S, Al-Aly Z. Estimated GFR Trajectories of People Entering CKD Stage 4 and Subsequent Kidney Disease Outcomes and Mortality. Am J Kidney Dis. 2016;68(2):219–28. doi: 10.1053/j.ajkd.2016.02.039 26948835

[5] Bruera S, Chisholm G, Dos Santos R, Crovador C, Bruera E, Hui D. Variations in vital signs in the last days of life in patients with advanced cancer. J Pain Symptom Manage. 2014;48(4):510–7. doi: 10.1016/j.jpainsymman.2013.10.019 24731412 PMC4197073

[6] Kubo Y. Changes in C-Reactive Protein, Albumin, and Hemoglobin in Breast Cancer Patients at the End-of-Life. Kitakanto Med J. 2019;69(2):105–10. doi: 10.2974/kmj.69.105

[7] Chesnaye NC, Caskey FJ, Dekker FW, de Rooij ENM, Evans M, Heimburger O, et al. Clinical and patient-reported trajectories at end-of-life in older patients with advanced CKD. Nephrol Dial Transplant. 2023;38(11):2494–502. doi: 10.1093/ndt/gfad091 37193666

[8] Boroujeni AM, Yousefi E, Zuretti A. Time-Series Analysis of Laboratory Values in the Context of Long-Term Hospitalized Patient Mortality. Am J Clin Pathol. 2019;151(5):452–60. doi: 10.1093/ajcp/aqy163 30689683

[9] Odagiri T, Watanabe H, Asai Y. A Retrospective Observational Study to Explore Trajectories of Hematologic Data and Palliative Performance Scale Scores in the Last 12 Weeks among Patients with Terminal-stage Cancer. Palliat Care Res. 2018;13(4):329–34. doi: 10.2512/jspm.13.329

[10] Suh S-Y, Ahn H-Y. Lactate dehydrogenase as a prognostic factor for survival time of terminally ill cancer patients: a preliminary study. Eur J Cancer. 2007;43(6):1051–9. doi: 10.1016/j.ejca.2007.01.031 17349786

[11] Masman AD, Tibboel D, Baar FPM, van Dijk M, Mathot RAA, van Gelder T. Prevalence and Implications of Abnormal Laboratory Results in Patients in the Terminal Phase of Life. J Palliat Med. 2016;19(8):822–9. doi: 10.1089/jpm.2015.0548 27494223

[12] Hui D, dos Santos R, Chisholm G, Bansal S, Silva TB, Kilgore K, et al. Clinical signs of impending death in cancer patients. Oncologist. 2014;19(6):681–7. doi: 10.1634/theoncologist.2013-0457 24760709 PMC4041673

[13] Herrinton LJ, Neslund-Dudas C, Rolnick SJ, Hornbrook MC, Bachman DJ, Darbinian JA, et al. Complications at the end of life in ovarian cancer. J Pain Symptom Manage. 2007;34(3):237–43. doi: 10.1016/j.jpainsymman.2006.11.011 17606360

[14] Fernandez FG, Kosinski AS, Furnary AP, Onaitis M, Kim S, Habib RH, et al. Differential effects of operative complications on survival after surgery for primary lung cancer. J Thorac Cardiovasc Surg. 2018;155(3):1254-1264.e1. doi: 10.1016/j.jtcvs.2017.09.149 29221736

[15] Dixon MR, Haukoos JS, Udani SM, Naghi JJ, Arnell TD, Kumar RR, et al. Carcinoembryonic antigen and albumin predict survival in patients with advanced colon and rectal cancer. Arch Surg. 2003;138(9):962–6. doi: 10.1001/archsurg.138.9.962 12963652

[16] Kawakami E, Tabata J, Yanaihara N, Ishikawa T, Koseki K, Iida Y, et al. Application of Artificial Intelligence for Preoperative Diagnostic and Prognostic Prediction in Epithelial Ovarian Cancer Based on Blood Biomarkers. Clin Cancer Res. 2019;25(10):3006–15. doi: 10.1158/1078-0432.CCR-18-3378 30979733

[17] Landi I, Glicksberg BS, Lee H-C, Cherng S, Landi G, Danieletto M, et al. Deep representation learning of electronic health records to unlock patient stratification at scale. NPJ Digit Med. 2020;3:96. doi: 10.1038/s41746-020-0301-z 32699826 PMC7367859

[18] Lundberg SM, Lee S-I. A unified approach to interpreting model predictions. Adv Neural Inf Process Syst. 2017;30.

[19] Moncada-Torres A, van Maaren MC, Hendriks MP, Siesling S, Geleijnse G. Explainable machine learning can outperform Cox regression predictions and provide insights in breast cancer survival. Sci Rep. 2021;11(1):6968. doi: 10.1038/s41598-021-86327-7 33772109 PMC7998037

[20] Bibault J-E, Hancock S, Buyyounouski MK, Bagshaw H, Leppert JT, Liao JC, et al. Development and Validation of an Interpretable Artificial Intelligence Model to Predict 10-Year Prostate Cancer Mortality. Cancers (Basel). 2021;13(12):3064. doi: 10.3390/cancers13123064 34205398 PMC8234681

[21] Lu S, Chen R, Wei W, Belovsky M, Lu X. Understanding Heart Failure Patients EHR Clinical Features via SHAP Interpretation of Tree-Based Machine Learning Model Predictions. AMIA Annu Symp Proc. 2022;2021:813–22. 35308970 PMC8861751

[22] Rodríguez-Belenguer P, Piñana JL, Sánchez-Montañés M, Soria-Olivas E, Martínez-Sober M, Serrano-López AJ. A machine learning approach to identify groups of patients with hematological malignant disorders. Comput Methods Programs Biomed. 2024;246:108011. doi: 10.1016/j.cmpb.2024.108011 38325024

[23] Ngusie HS, Mengiste SA, Zemariam AB, Molla B, Tesfa GA, Seboka BT, et al. Predicting adverse birth outcome among childbearing women in Sub-Saharan Africa: employing innovative machine learning techniques. BMC Public Health. 2024;24(1):2029. doi: 10.1186/s12889-024-19566-8 39075434 PMC11285398

[24] Sakuragi M, Uchino E, Sato N, Matsubara T, Ueda A, Mineharu Y, et al. Interpretable machine learning-based individual analysis of acute kidney injury in immune checkpoint inhibitor therapy. PLoS One. 2024;19(3):e0298673. doi: 10.1371/journal.pone.0298673 38502665 PMC10950216

[25] Ke G, Meng Q, Finley T, Wang T, Chen W, Ma W. Lightgbm: A highly efficient gradient boosting decision tree. Adv Neural Inf Process Syst. 2017;30.

[26] Akiba T, Sano S, Yanase T, Ohta T, Koyama M. Optuna: a next-generation hyperparameter optimization framework. In: Proceedings of the 25th ACM SIGKDD International Conference on Knowledge Discovery & Data Mining, 2019. 2623–31. doi: 10.1145/3292500.3330701

[27] McInnes L, Healy J, Melville J. Umap: uniform manifold approximation and projection for dimension reduction. ArXiv. 2018. doi: 10.48550/arXiv.1802.03426

[28] Van der Maaten L, Hinton G. Visualizing data using t-SNE. J Mach Learn Res. 2008;9:2579–605.

[29] Cheng L, DeJesus AY, Rodriguez MA. Using Laboratory Test Results at Hospital Admission to Predict Short-term Survival in Critically Ill Patients With Metastatic or Advanced Cancer. J Pain Symptom Manage. 2017;53(4):720–7. doi: 10.1016/j.jpainsymman.2016.11.008 28062337

[30] Suh S-Y, Ahn H-Y. A prospective study on C-reactive protein as a prognostic factor for survival time of terminally ill cancer patients. Support Care Cancer. 2007;15(6):613. doi: 10.1007/s00520-006-0208-5 17235502

[31] Amano K, Maeda I, Morita T, Miura T, Inoue S, Ikenaga M, et al. Clinical Implications of C-Reactive Protein as a Prognostic Marker in Advanced Cancer Patients in Palliative Care Settings. J Pain Symptom Manage. 2016;51(5):860–7. doi: 10.1016/j.jpainsymman.2015.11.025 26826676

[32] Ho S-Y, Guo H-R, Chen HHW, Peng C-J. Nutritional predictors of survival in terminally ill cancer patients. J Formos Med Assoc. 2003;102(8):544–50. 14569319

[33] Gray S, Axelsson B. The prevalence of deranged C-reactive protein and albumin in patients with incurable cancer approaching death. PLoS One. 2018;13(3):e0193693. doi: 10.1371/journal.pone.0193693 29534089 PMC5849305

[34] Evans WJ, Morley JE, Argilés J, Bales C, Baracos V, Guttridge D, et al. Cachexia: a new definition. Clin Nutr. 2008;27(6):793–9. doi: 10.1016/j.clnu.2008.06.013 18718696

