# Supplementary material for: Predicting mortality dynamics in cancer patients: A machine learning approach to pre-death events
Source: PLoS One. 2025 Sep 9;20(9):e0331650. doi: 10.1371/journal.pone.0331650 (PMC12419616; doi:10.1371/journal.pone.0331650)
Supplement: S1 Text — S1 File. Supplemental information of methodology. S2 File. Laboratory parameter list. S3 File. Performances and confusion matrices of continuous mortality prediction models. S4 File. Mean SHAP values of all parameters immediately before death. S5 File. Reference values of ALB, CRP, BUN, and LDH. S6 File. Details of visualizing changes in patient states using time-series SHAP values. S7 File. Evaluation of the number of clusters in patient stratification using SHAP values. S8 File. Stratification of patient states using laboratory values. S9 File. SHAP behaviors of the top influential items for each subtype. S10 File. Statistical tests on laboratory test values, biological sex, age, and cancer type. S11 File. Detailed analysis and discussion of the background of the patient state change subtypes. (ZIP) [file pone.0331650.s001.zip › supplemental_data_20250407/supplemental_data_s6.docx]

**Supplemental Data S6 Details of visualizing changes in patient states using time-series SHAP values**

Missing values complicates dimensionality reduction. Therefore, we extracted only the features with a missing rate of 10% or less at all time points from 1 day to 90 days before death. By removing patient samples with missing values in these features, we created a dataset without missing values. The extracted features are shown in Table S6-1. All top 10 significant test items, except eosinophil, were included, and all features were blood parameters.


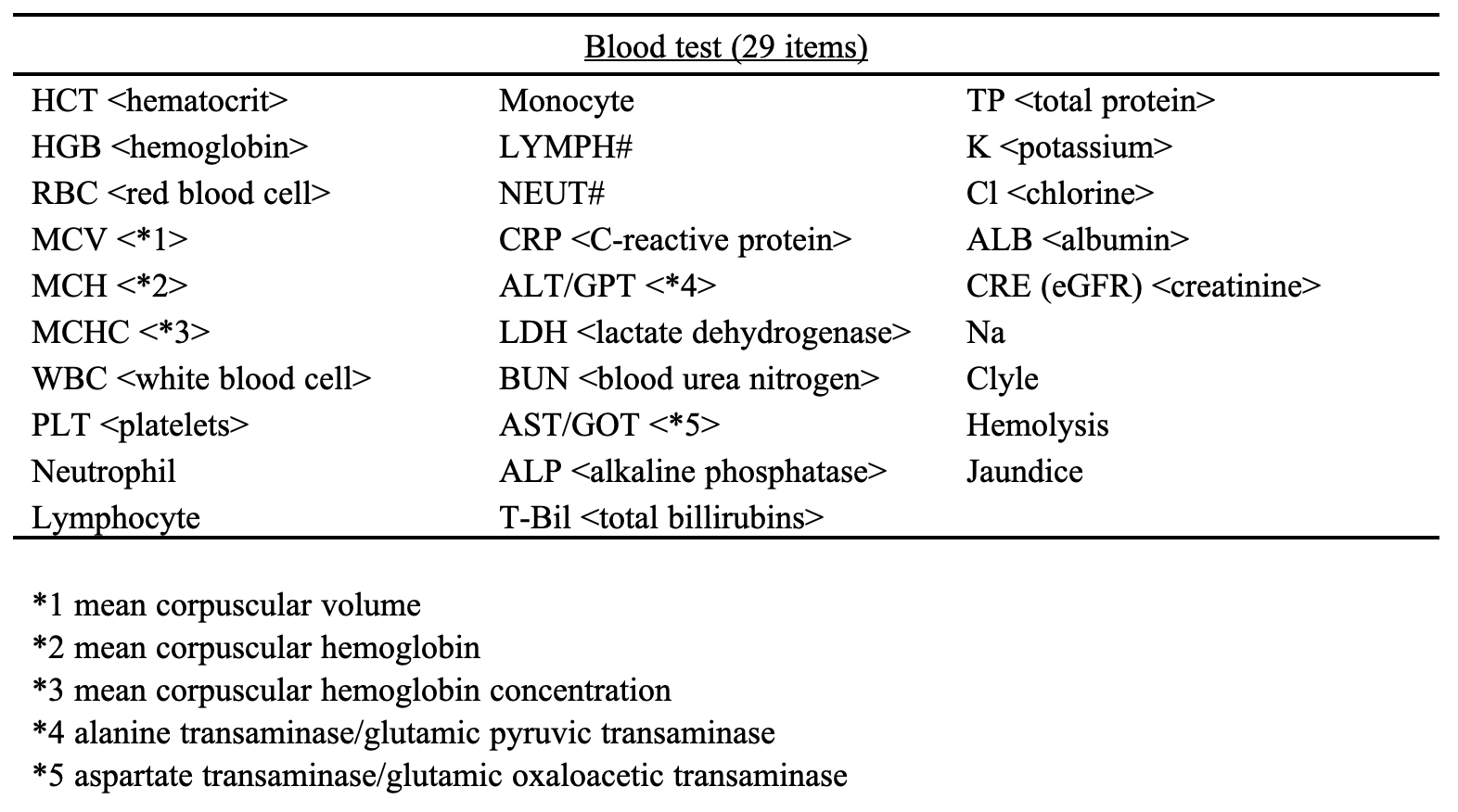


**Table S6-1. Laboratory test items extracted as features for dimensionality reduction.**

Fig S6-1A is the visualization of the SHAP trajectory using t-SNE and PCA. Similar to the trajectory visualized by UMAP (Fig 3), both methods were able to clearly capture temporal changes solely based on SHAP values. This suggests that regardless of the methods, SHAP values are more suitable for capturing temporal changes in patient state than raw laboratory test values.

**
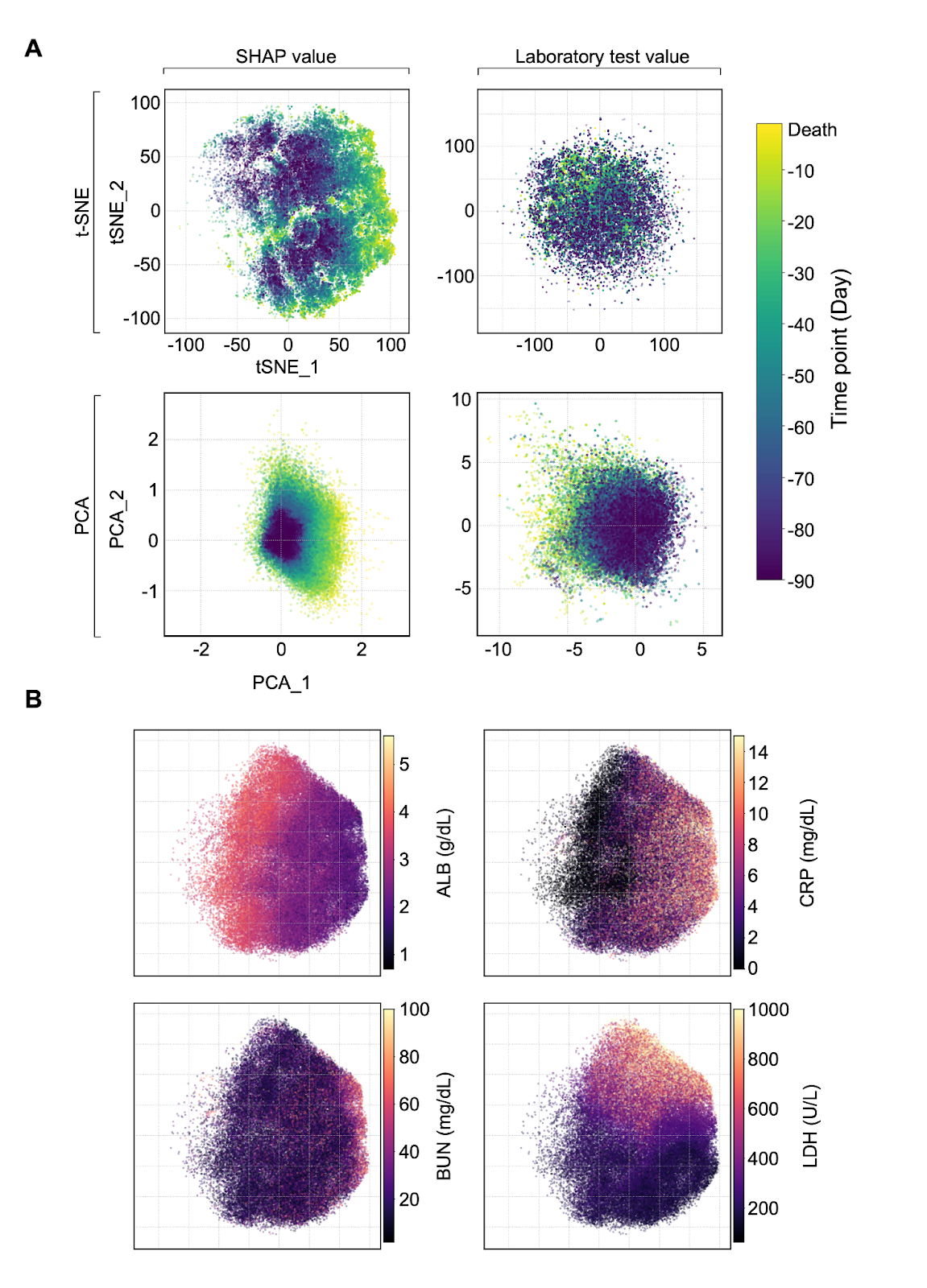
**

**Fig S6-1. Visualization of the SHAP trajectory by t-SNE and PCA, and correspondence to laboratory values.**

**(A)** Visualization of the SHAP trajectory using (Top) t-SNE (bottom) PCA. Dimensionality reduction results to 2D based on (Left) SHAP values (Right) laboratory test values. The color bar indicates the number of days before death. **(B)** Distribution of ALB, CRP, BUN, and LDH test values in the visualization based on UMAP using SHAP values (Fig 3).

Additionally, for the four particularly important test items among the top 10 significant test items, namely ALB, CRP, LDH, and BUN, we present in Fig S6-1B the distribution of each laboratory test value corresponding to the UMAP dimension reduction results based on SHAP values (Fig 3). ALB showed smaller values towards the right side of the distribution, while CRP and BUN exhibited larger values towards the right side. LDH had larger values towards the upper part of the distribution. Thus, significant differences were observed in the distribution trends for each test item, indicating varied test value patterns across different parts of the distribution.

As observed in Fig 3 and Fig S6-1A, the widening distribution closer to death in the visualizations based on SHAP values is considered to be related with the overall increase in SHAP values. Specifically, it could be said that SHAP values for each test item are smaller and differences between SHAP value patterns are less pronounced at points further from death, leading to relatively dense distributions.
