# Supplementary material for: Predicting mortality dynamics in cancer patients: A machine learning approach to pre-death events
Source: PLoS One. 2025 Sep 9;20(9):e0331650. doi: 10.1371/journal.pone.0331650 (PMC12419616; doi:10.1371/journal.pone.0331650)
Supplement: S1 Text — S1 File. Supplemental information of methodology. S2 File. Laboratory parameter list. S3 File. Performances and confusion matrices of continuous mortality prediction models. S4 File. Mean SHAP values of all parameters immediately before death. S5 File. Reference values of ALB, CRP, BUN, and LDH. S6 File. Details of visualizing changes in patient states using time-series SHAP values. S7 File. Evaluation of the number of clusters in patient stratification using SHAP values. S8 File. Stratification of patient states using laboratory values. S9 File. SHAP behaviors of the top influential items for each subtype. S10 File. Statistical tests on laboratory test values, biological sex, age, and cancer type. S11 File. Detailed analysis and discussion of the background of the patient state change subtypes. (ZIP) [file pone.0331650.s001.zip › supplemental_data_20250407/supplemental_data_s7.docx]

**Supplemental Data S7 Evaluation of the number of clusters in patient stratification using SHAP values**

In order to determine the optimal number of clusters for stratification of patients one day before death using SHAP values, evaluation using the silhouette score was performed (Fig S7-1A). The silhouette score was highest when the number of clusters was two, followed by three. In the ELBOW evaluation, it was difficult to determine the optimum number of clusters based on the SSE (Sum of Squared Error) trend (Fig S7-1B). When the number of clusters was two, the optimal number of clusters was determined to be three, since there was a large difference in sample size among the clusters.


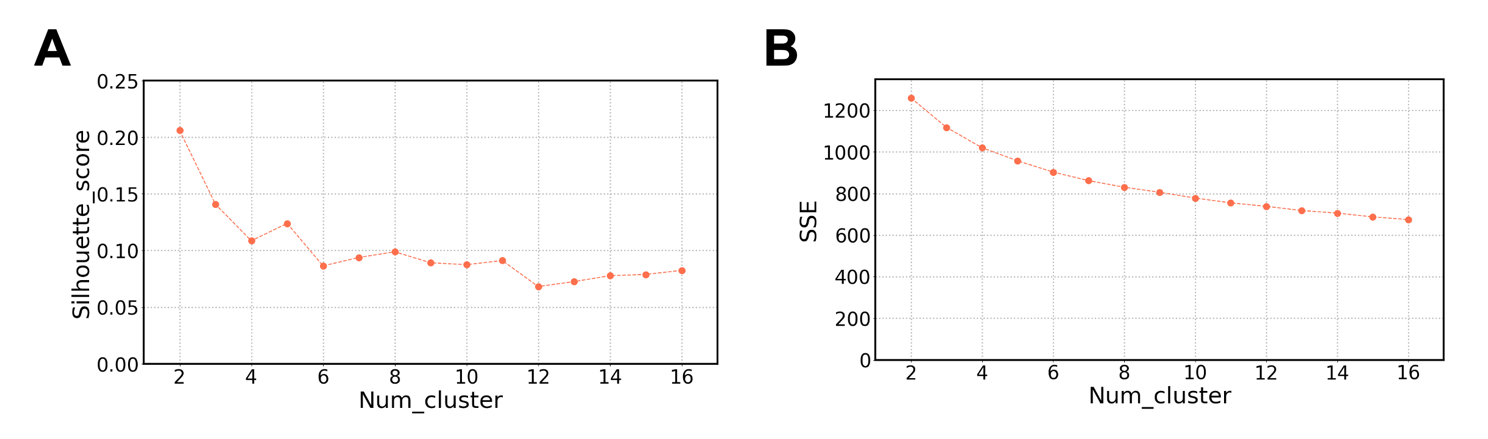


**Fig S7-1. Results of the Silhouette and ELBOW methods.**

**(A)** Horizontal axis shows number of clusters and vertical axis shows silhouette score. **(B)** Horizontal and vertical axes indicate the number of clusters and SSE, respectively.
