# Supplementary material for: Predicting mortality dynamics in cancer patients: A machine learning approach to pre-death events
Source: PLoS One. 2025 Sep 9;20(9):e0331650. doi: 10.1371/journal.pone.0331650 (PMC12419616; doi:10.1371/journal.pone.0331650)
Supplement: S1 Text — S1 File. Supplemental information of methodology. S2 File. Laboratory parameter list. S3 File. Performances and confusion matrices of continuous mortality prediction models. S4 File. Mean SHAP values of all parameters immediately before death. S5 File. Reference values of ALB, CRP, BUN, and LDH. S6 File. Details of visualizing changes in patient states using time-series SHAP values. S7 File. Evaluation of the number of clusters in patient stratification using SHAP values. S8 File. Stratification of patient states using laboratory values. S9 File. SHAP behaviors of the top influential items for each subtype. S10 File. Statistical tests on laboratory test values, biological sex, age, and cancer type. S11 File. Detailed analysis and discussion of the background of the patient state change subtypes. (ZIP) [file pone.0331650.s001.zip › supplemental_data_20250407/supplemental_data_s10.docx]

**Supplemental Data S10 Statistical tests on laboratory test values, biological sex, age, and cancer type**

To investigate whether there are differences in laboratory test values among patient state subtypes stratified using SHAP values from one day before death, we examined the top 10 influential parameters (ALB, CRP, BUN, LDH, Lymphocyte, Cl, WBC, Neutrophil, TP, Eosinophil), excluding Eosinophil which did not meet the criteria described in the Method (also described in S6 Appendix). The Kruskal-Wallis test was conducted at a 1% significance level across all subtypes (Table S10-1). The null hypothesis was rejected for ALB, CRP, BUN, Cl and TP (p < 0.01).

| Laboratory test item | *p*-value |  | Laboratory test item | *p*-value |
| --- | --- | --- | --- | --- |
| ALB | **< 0.01** |  | Cl | **< 0.01** |
| CRP | **< 0.01** |  | WBC | 0.05 |
| BUN | **< 0.01** |  | Neutrophil | 0.74 |
| LDH | 0.03 |  | TP | **< 0.01** |
| Lymphocyte | 0.50 |  |  |  |

**Table S10-1. Results of Kruskal-Wallis test.**

Test results for difference of population mean against laboratory test values one day before death. p-values for ALB, CRP, BUN, Cl and TP were less than the significance level.

Then, except for Lymphocyte, we verified the differences between individual subtypes using the Mann-Whitney U test. In this multiple comparison, Bonferroni correction was applied for a significance level of 1%. The results are shown in Table S10-2. For ALB, subtype 1 had significantly higher values compared to the other subtypes (p < 0.001). For CRP, subtype 2 had significantly lower (p < 0.001) and subtype 3 had significantly higher (p < 0.001) values compared to the other subtypes. For BUN, subtype 1 had significantly lower (p < 0.001) and subtype 2 had significantly higher (p < 0.001) values compared to the other subtypes. For Cl, subtype 1 had significantly lower (p < 0.001) and subtype 2 had significantly higher (p < 0.001) values compared to the other subtypes. For TP, subtype 1 had significantly higher (p < 0.001) and subtype 2 had significantly lower (p < 0.0033) values compared to the other subtypes.

|  | p-value |  | p-value |
| --- | --- | --- | --- |
| **ALB** |  | **Cl** |  |
| Subtype 1-2 | **< 0.001** | Subtype 1-2 | **< 0.001** |
| Subtype 1-3 | **< 0.001** | Subtype 1-3 | **< 0.001** |
| Subtype 2-3 | 0.512 | Subtype 2-3 | **< 0.001** |
| **CRP** |  | **TP** |  |
| Subtype 1-2 | **< 0.001** | Subtype 1-2 | **< 0.001** |
| Subtype 1-3 | **< 0.001** | Subtype 1-3 | **< 0.001** |
| Subtype 2-3 | **< 0.001** | Subtype 2-3 | **0.001** |
| **BUN** |  |  |  |
| Subtype 1-2 | **< 0.001** |  |  |
| Subtype 1-3 | **< 0.001** |  |  |
| Subtype 2-3 | **< 0.001** |  |  |

**Table S10-2. Results of Mann-Whitney U test.**

The significance level was adjusted for multiple comparisons by comparing the p-values against 1×10^-2^/3 (≈ 0.0033), given that three comparisons were conducted for each parameter.

Next, we aggregated data on biological sex, age group, and cancer type classification based on ICD10 codes for each patient subtype and analyzed whether there were differences in these proportions across subtypes. We conducted a chi-squared test with a significance level of 1% for all subtypes (Table S10-3). If the null hypothesis was rejected, we verified differences between individual subtypes using Tukey's multiple comparison test at a significance level of 1% (Table S10-4). For age, the subtype 1 proportion was significantly lower than all other subtypes in the 60-79 age group (p < 0.01). For cancer type classification, the subtype 2 proportion of lymphoid, hematopoietic, and related tissue were significantly higher than subtype 3 (p < 0.01).

|  | Subtype 1 | Subtype 2 | Subtype 3 | *p*-value |
| --- | --- | --- | --- | --- |
| **Patients** | 114 | 65 | 367 | ― |
| **Sex** |  |  |  |  |
| Male | 41 (35.96) | 25 (38.46) | 138 (37.60) | 0.933 |
| Female | 73 (64.04) | 40 (61.54) | 229 (62.40) | ― |
| **Age** |  |  |  |  |
| 20-39 | 9 (7.89) | 4 (6.15) | 16 (4.36) | 0.322 |
| 40-59 | 40 (35.09) | 12 (18.46) | 86 (23.43) | 0.017 |
| 60-79 | 54 (47.37) | 46 (70.77) | 235 (64.03) | **< 0.01** |
| 80 and above | 11 (9.65) | 3 (4.62) | 30 (8.17) | 0.488 |
| **Cancer type (ICD-10)** |  |  |  |  |
| Lip, oral cavity, and pharynx | 0 (0) | 1 (1.54) | 8 (2.18) | 0.279 |
| Digestive organs | 32 (28.07) | 13 (20.00) | 105 (28.61) | 0.353 |
| Respiratory and intrathoracic organs | 16 (14.04) | 6 (9.23) | 27 (7.36) | 0.093 |
| Bone and articular cartilage | 2 (1.75) | 1 (1.54) | 0 (0) | 0.045 |
| Melanoma and skin | 0 (0) | 0 (0) | 2 (0.54) | 0.613 |
| Mesothelial and soft tissue | 0 (0) | 0 (0) | 5 (1.36) | 0.292 |
| Breast | 2 (1.75) | 1 (1.54) | 5 (1.36) | 0.954 |
| Female genital organs | 4 (3.51) | 1 (1.54) | 17 (4.63) | 0.480 |
| Male genital organs | 2 (1.75) | 0 (0) | 12 (3.27) | 0.254 |
| Urinary tract | 3 (2.63) | 0 (0) | 15 (4.09) | 0.213 |
| Eye, brain, and other parts of CNS | 0 (0) | 0 (0) | 2 (0.54) | 0.613 |
| Thyroid and other endocrine glands | 2 (1.75) | 0 (0) | 0 (0) | 0.022 |
| Secondary and unspecified sites | 24 (21.05) | 11 (16.92) | 79 (21.53) | 0.701 |
| Lymphoid, hematopoietic, and related tissue | 19 (16.67) | 23 (35.38) | 68 (18.53) | **< 0.01** |
| In situ neoplasms | 0 (0) | 0 (0) | 0 (0) | - |
| Benign neoplasms | 2 (1.75) | 4 (6.15) | 9 (2.45) | 0.186 |
| Uncertain or unknown behavior | 6 (5.26) | 4 (6.15) | 13 (3.54) | 0.515 |

**Table S10-3. Statistical results for sex, age group, and cancer type classification in each subtype.**

Cancer types are based on ICD10 codes. The numbers in the table are presented in the format of counts (percentages), and ** indicates α<.01. The p-values represent the results of the chi-squared test.

|  | WSD | P | Result |
| --- | --- | --- | --- |
| **Age group 60-79** |  |  |  |
| Subtype 1 - 2 | 0.228 | 0.234 | ** |
| Subtype 1 - 3 | 0.149 | 0.167 | ** |
| Subtype 2 - 3 | 0.182 | 0.067 | - |
| **ICD-10: Lymphoid, hematopoietic, and related tissue** |  |  |  |
| Subtype 1 - 2 | 0.188 | 0.188 | - |
| Subtype 1 - 3 | 0.117 | 0.019 | - |
| Subtype 2 - 3 | 0.156 | 0.169 | ** |

**Table S10-4. Results of Tukey’s multiple comparison tests.**

The P column represents the difference in frequency between compared subtypes. If WSD is smaller than P, the null hypothesis is rejected. The Result column indicates ** for significance at the 1% level, and - for no significance.
