# Supplementary material for: Predicting mortality dynamics in cancer patients: A machine learning approach to pre-death events
Source: PLoS One. 2025 Sep 9;20(9):e0331650. doi: 10.1371/journal.pone.0331650 (PMC12419616; doi:10.1371/journal.pone.0331650)
Supplement: S1 Text — S1 File. Supplemental information of methodology. S2 File. Laboratory parameter list. S3 File. Performances and confusion matrices of continuous mortality prediction models. S4 File. Mean SHAP values of all parameters immediately before death. S5 File. Reference values of ALB, CRP, BUN, and LDH. S6 File. Details of visualizing changes in patient states using time-series SHAP values. S7 File. Evaluation of the number of clusters in patient stratification using SHAP values. S8 File. Stratification of patient states using laboratory values. S9 File. SHAP behaviors of the top influential items for each subtype. S10 File. Statistical tests on laboratory test values, biological sex, age, and cancer type. S11 File. Detailed analysis and discussion of the background of the patient state change subtypes. (ZIP) [file pone.0331650.s001.zip › supplemental_data_20250407/supplemental_data_s1.docx]

**Supplemental Data S1 Supplemental information of methodology**

**
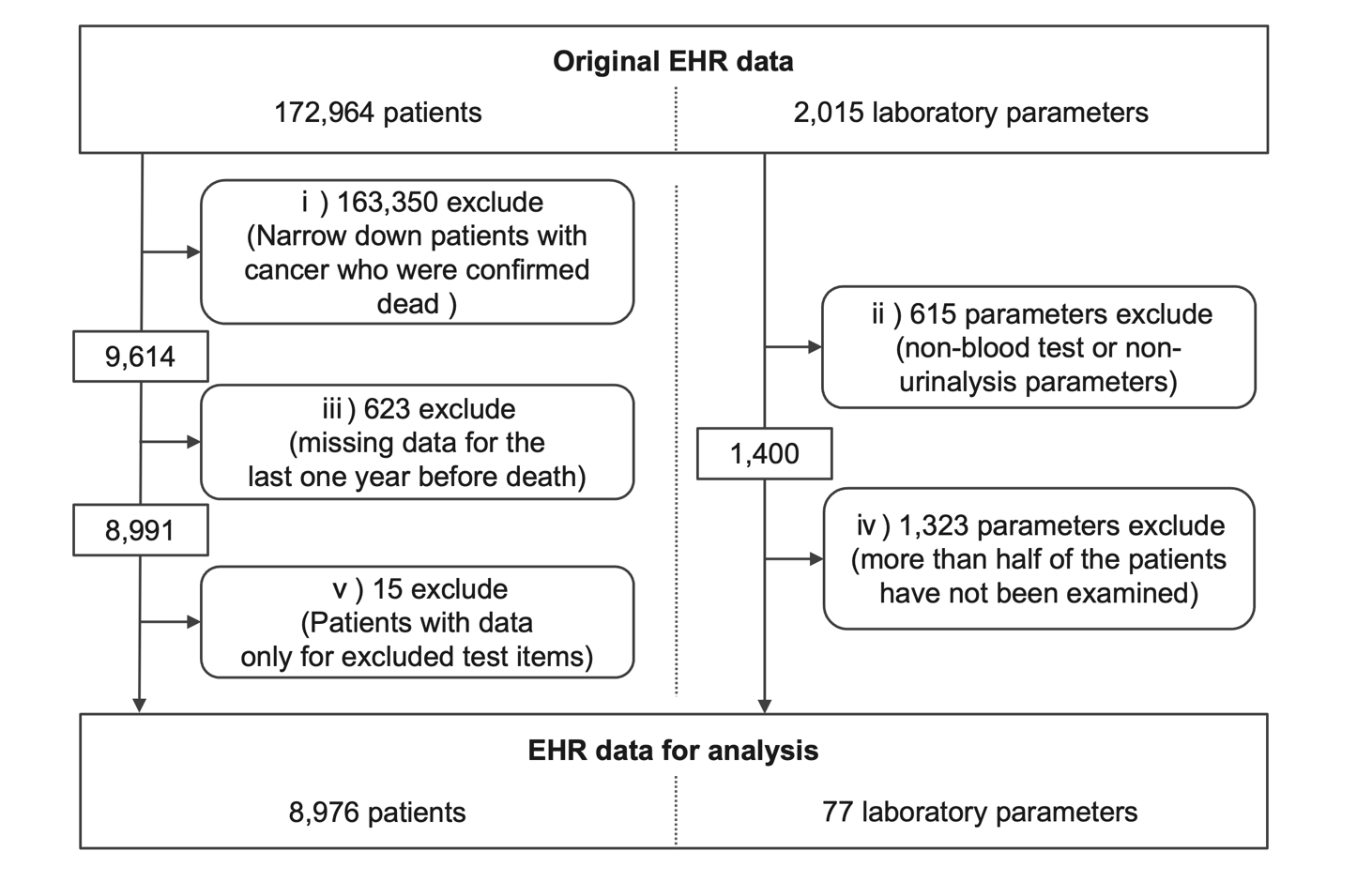
**

**Fig S1-1. Data preprocessing procedures.**

The panel on the left side of the dotted line illustrates the patient selection flow. The panel on the right side depicts the selection of laboratory parameters. Roman numerals indicate the processing steps.

**
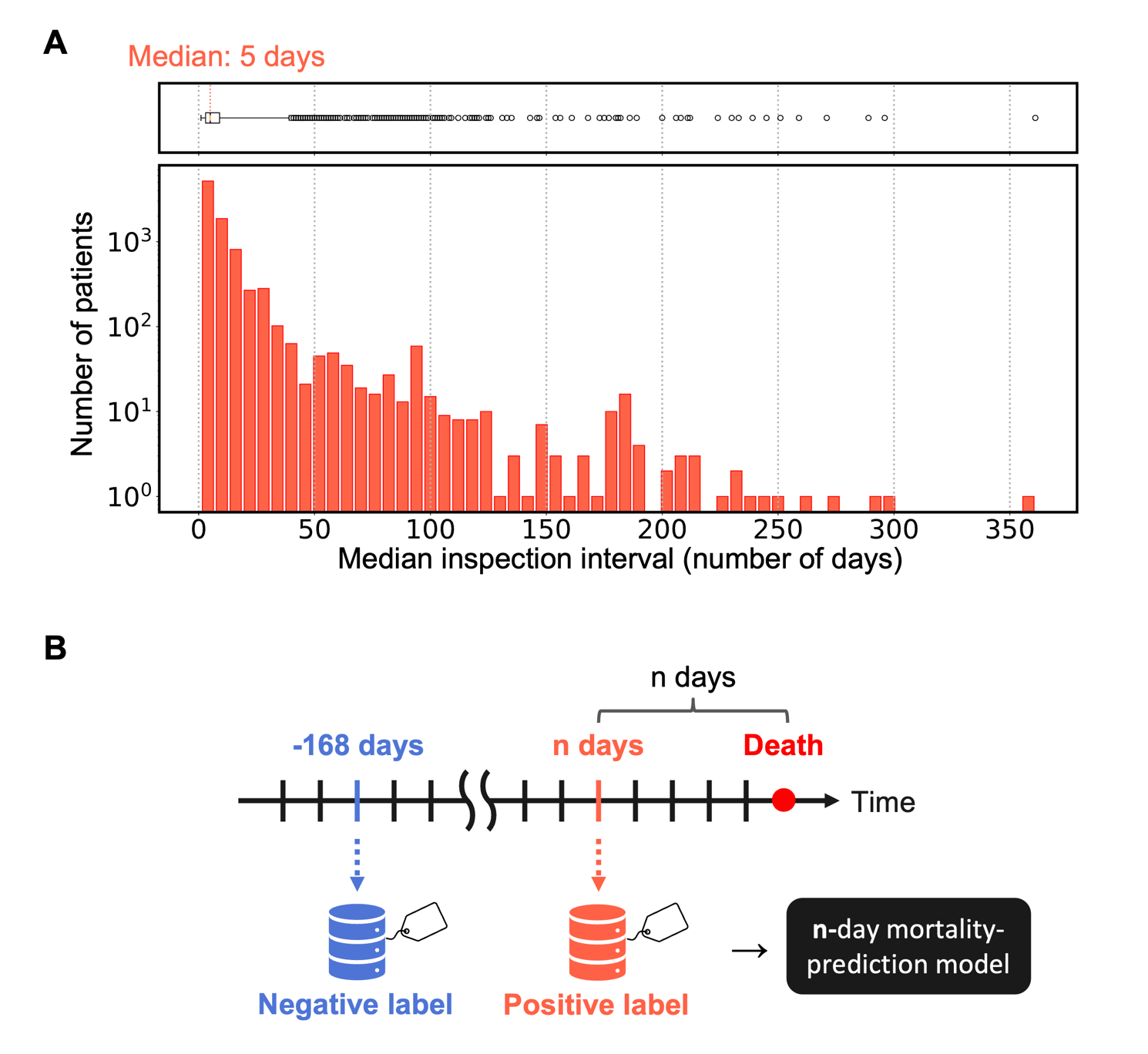
**

**Fig S1-2. Resampling and labeling method.**

**(A)** The distribution of median days between laboratory tests for each patient. The vertical axis of the histogram is shown in the log scale. The upper box plot corresponds to the histogram, with whiskers spanning the 5th to 95th percentile data. The median time based on the distribution was 5 days, which was selected as the width for resampling. Outliers are represented by circles. **(B)** Labeling of datasets for mortality-prediction models. To build the model predicting death “n” days later, positive labels were assigned to the laboratory test data obtained “n” days before death. For prediction models at any time point, negative labels were assigned to the laboratory test data from 168 days before death.

**
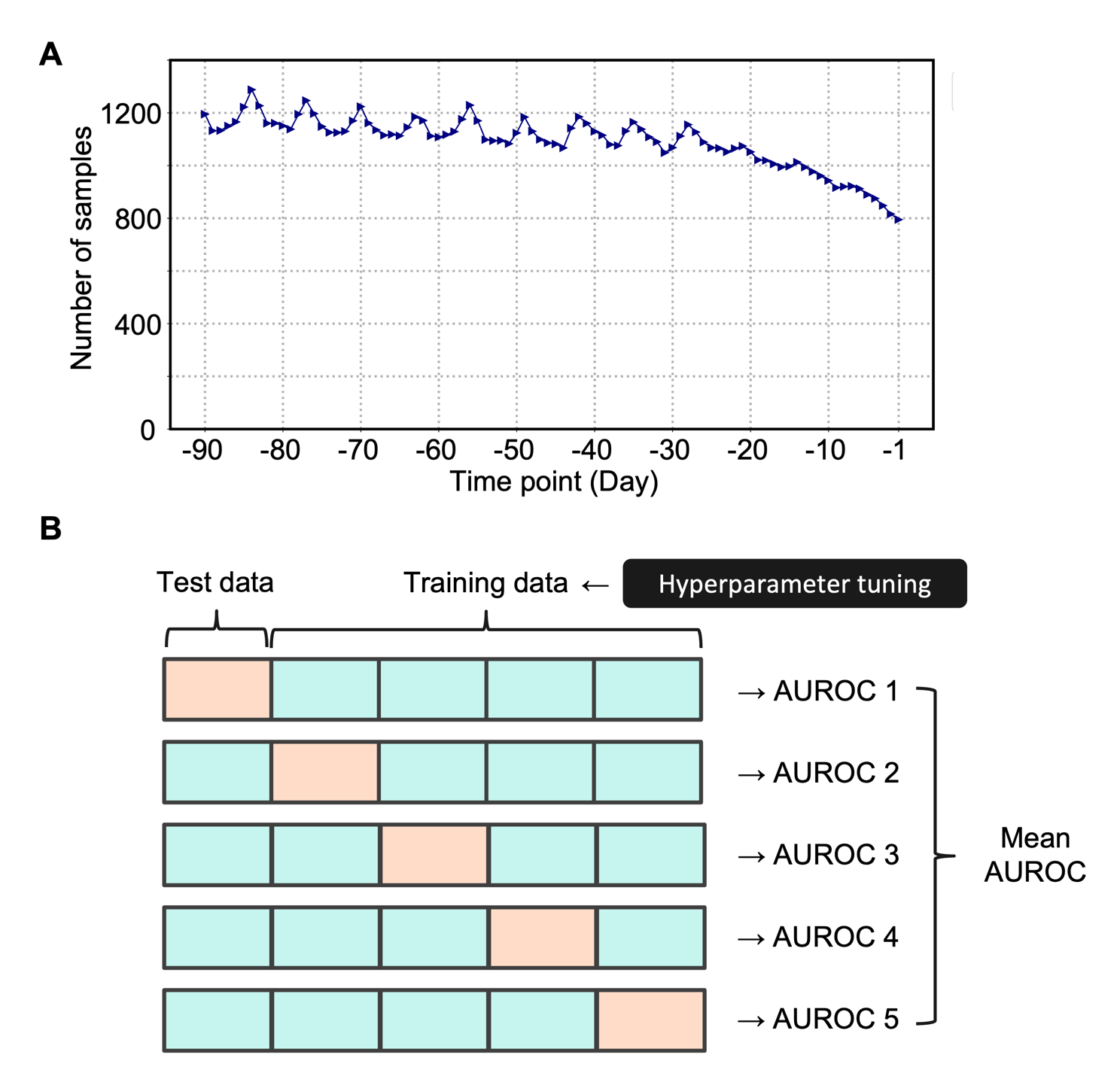
**

**Fig S1-3. Patient sample size at each time point and cross-validation method.**

(A) The number of patient samples at each time point. (B) Five-fold cross-validation. AUROC was calculated for each fold, and the mean of these values was used as the AUROC for evaluating the model constructed for that time point. Hyperparameter tuning was conducted using LightGBMTunerCV with the training data.

**
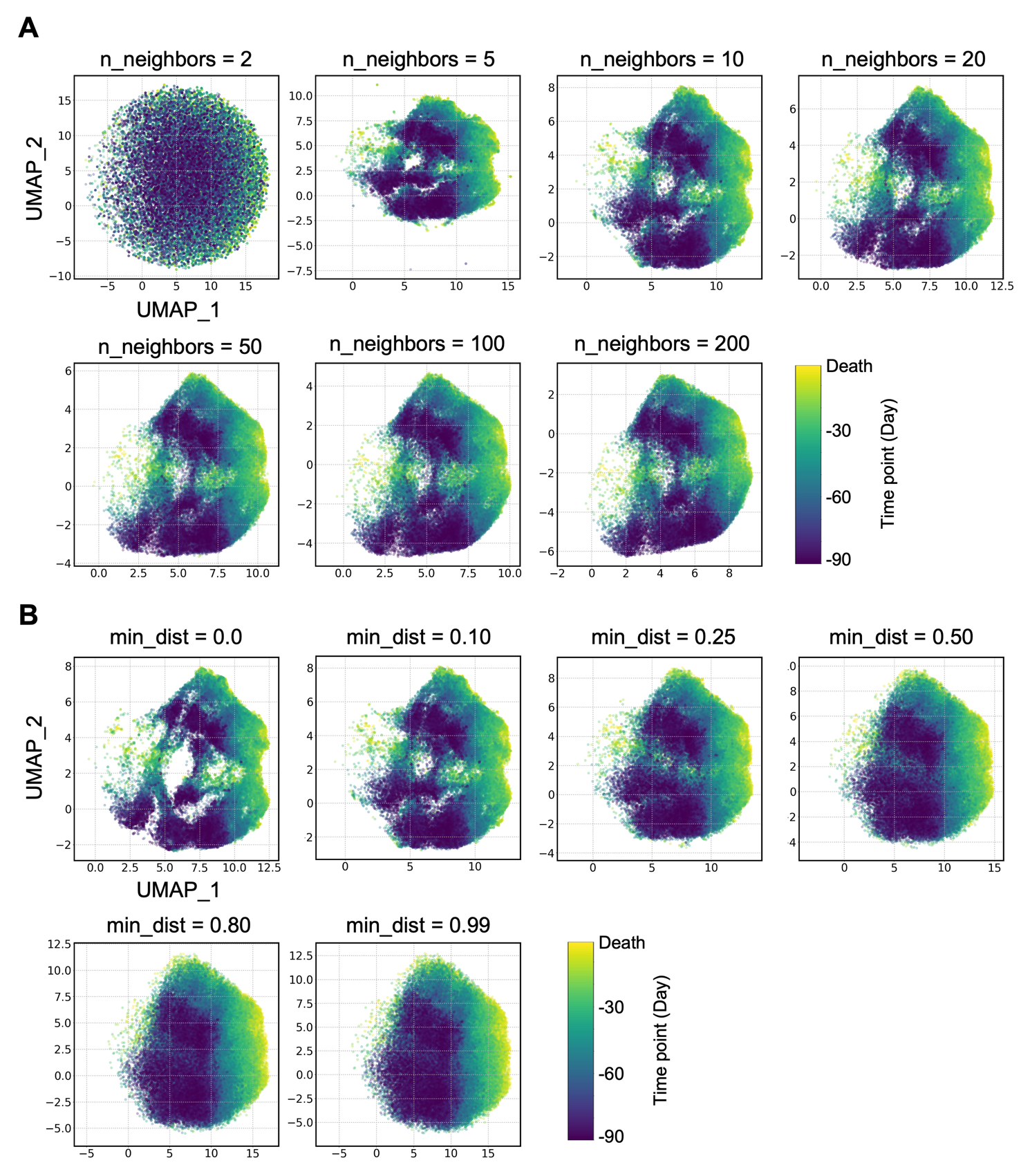
**

**Fig S1-4. UMAP plots for each hyperparameter.**

Changes in UMAP plots with hyperparameter adjustments. The color bar indicates the number of days before death, changing from navy to yellow with progressing time. The Euclidean distance metric was used to generate the plots, and the 'random_seed' was conventionally set to 0. (A) UMAP plots when adjusting the value of 'n_neighbors’ with 'min_dist' fixed at 0.10. Smaller values of the 'n_neighbors' captured more local structures, while larger values provided a more comprehensive representation of the overall data structure. (B). UMAP plots when the 'min_dist’ value was adjusted with 'n_neighbors' fixed at 10. Smaller values of the 'min_dist' parameter resulted in higher density of the data points in the plot.


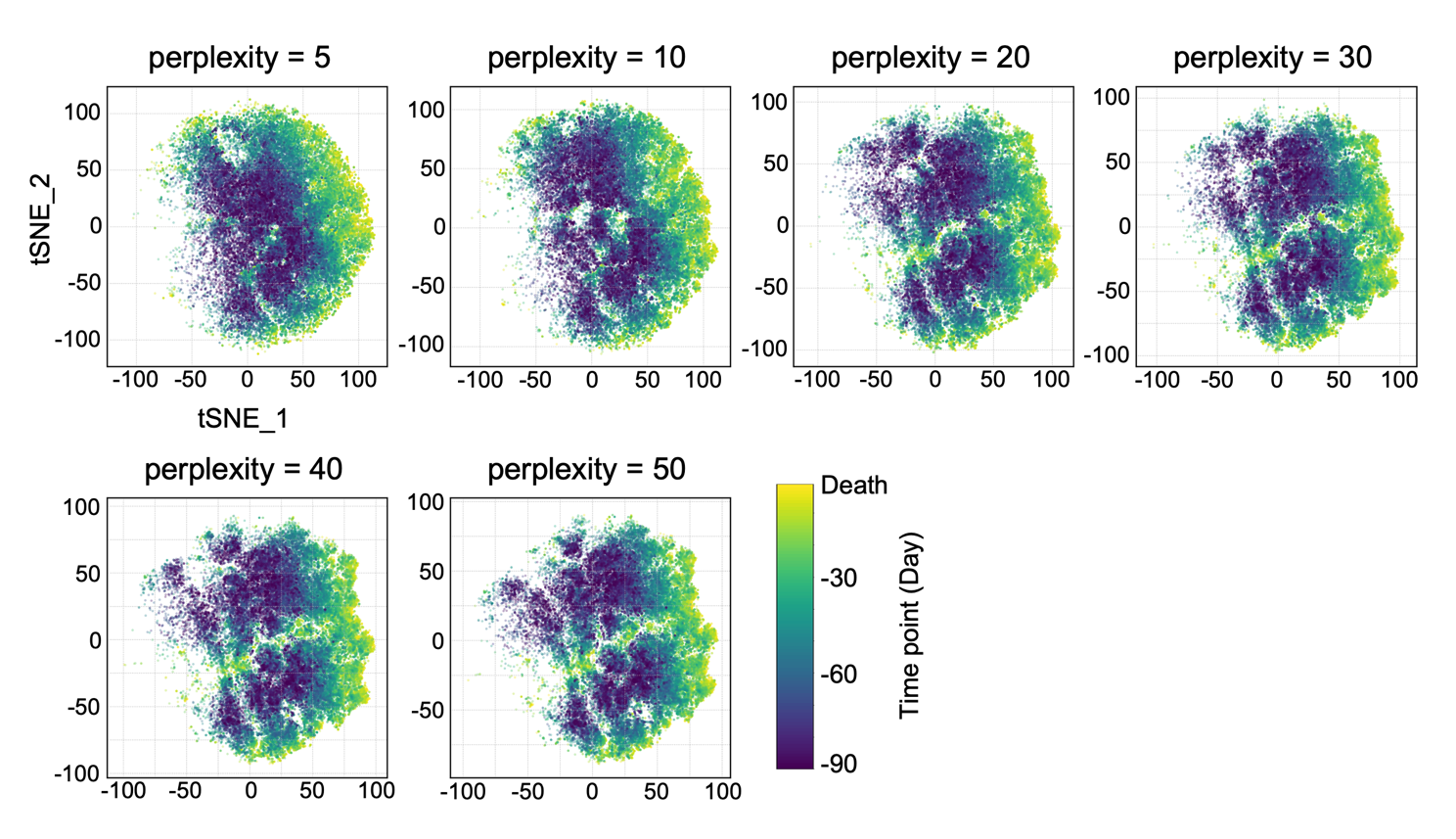


**Fig S1-5. t-SNE plots for each hyperparameter.**

Changes in t-SNE plots with hyperparameter adjustments. The value of 'random_state' was set to 0 in all cases, and 'n_neighbors' was fixed at 20. The 'perplexity' parameter determines whether the local or global structure of the data is prioritized; however, no apparent changes were observed between the plots.
