# Supplementary material for: Predicting mortality dynamics in cancer patients: A machine learning approach to pre-death events
Source: PLoS One. 2025 Sep 9;20(9):e0331650. doi: 10.1371/journal.pone.0331650 (PMC12419616; doi:10.1371/journal.pone.0331650)
Supplement: S1 Text — S1 File. Supplemental information of methodology. S2 File. Laboratory parameter list. S3 File. Performances and confusion matrices of continuous mortality prediction models. S4 File. Mean SHAP values of all parameters immediately before death. S5 File. Reference values of ALB, CRP, BUN, and LDH. S6 File. Details of visualizing changes in patient states using time-series SHAP values. S7 File. Evaluation of the number of clusters in patient stratification using SHAP values. S8 File. Stratification of patient states using laboratory values. S9 File. SHAP behaviors of the top influential items for each subtype. S10 File. Statistical tests on laboratory test values, biological sex, age, and cancer type. S11 File. Detailed analysis and discussion of the background of the patient state change subtypes. (ZIP) [file pone.0331650.s001.zip › supplemental_data_20250407/supplemental_data_s11.docx]

**Supplemental Data S11 Detailed analysis and discussion of the background of the patient state change subtypes**

For trajectories leading to each patient state subtype (Fig 4 and Fig 5), we analyzed whether there were differences between trajectories concerning renal failure, presence of infection, and classification of gastrointestinal cancer sites. Significant differences were observed between some trajectories; however, no specific trajectory was found to have a significant difference compared to all other trajectories. Detailed results of the statistical tests are provided at the end of this supplemental file.

Fig 4, Fig 5, and Table 2 suggest the existence of multiple subtypes in the patient state of terminal cancer, each with significantly different developmental trajectories. Here, we discuss the trajectories by which three patient state subtypes emerge immediately before death.

In the trajectory of subtype 1, LDH had the greatest influence throughout the 90 days preceding death, with minimal involvement of ALB and CRP. Given LDH's elevation in tissue damage, the trajectory of subtype 1 likely followed a death trajectory influenced by tissue damage. Indeed, while ALB felled below reference values (S5 Appendix), it remained relatively higher immediately before death (Table 2).

In the trajectory of subtype 2, ALB was the most influential parameter throughout the 90 days before death. While there was a characteristic increase in BUN's contribution one month before death (Table 2), the incidence of renal failure known to cause BUN elevation was not higher than in other trajectories. The importance of CRP was considerably smaller than those of ALB and BUN, and given the laboratory test value of CRP just before death (Table 2), it is difficult to argue that inflammation or tissue damage is more severe in this trajectory. The trajectory of subtype 2 may not necessarily involve cachexia, but it likely follows a trajectory of deteriorating nutritional states due to some cause.

The trajectory of subtype 3 was characterized by ALB's highest influence, followed by the inflammatory protein CRP, with relatively low importance of LDH. The low ALB and LDH levels just before death (Table 2) reflected this. These findings align with traits of cancer cachexia, characterized by increased protein breakdown and inflammatory protein production. That is, the trajectory of subtype 3 might reflect severe cancer cachexia.

As discussed above, there are multiple trajectories leading to death in patients with terminal cancer. Currently, the Evans classification [1] is used as a diagnostic criterion for cancer cachexia, with CRP and ALB as the laboratory test parameters. There are reports associating this classification with IL-6 overexpression suppression in the CRP-high and ALB-low groups in colorectal cancer, leading to recovery of the overall states to a state before cancer cachexia onset [2]. This study identified the importance of LDH and BUN in addition to ALB and CRP in the patient states dynamics of terminal cancer. Incorporating LDH and BUN into the classification of terminal cancer patients is expected to more effectively classify patients and design appropriate treatment plans for each classification.

The details of the statistical tests mentioned at the beginning of this section are as follows. We investigated whether there were differences in the incidence frequencies of tumors in various digestive organs, renal failure, and infections along each trajectory leading to the subtypes. Frequencies were defined as the proportion of patients diagnosed with these states within one year before death relative to the total number of patients in each subtype. However, for infections, we specifically examined incidences within 60 days before death to focus on those occurring closer to the time of death. After aggregating the frequencies, we performed chi-square tests with a significance level set at 1% (Table S11-1).

|  | Subtype 1 | Subtype 2 | Subtype 3 | *p*-value |
| --- | --- | --- | --- | --- |
| **Patients** | 114 | 65 | 367 | ― |
| **Digestive organs** |  |  |  |  |
| Esophagus (E) | 1 (0.88) | 0 (0) | 11 (3.00) | 0.176 |
| Stomach (S) | 6 (5.26) | 4 (6.15) | 21 (5.72) | 0.968 |
| Small intestine | 1 (0.88) | 1 (1.54) | 1 (0.27) | 0.386 |
| Large intestine (L) | 4 (3.51) | 0 (0) | 17 (4.63) | 0.197 |
| Pancreas | 1 (0.88) | 2 (3.08) | 19 (5.18) | 0.042 |
| Liver | 9 (7.89) | 5 (7.69) | 44 (11.99) | 0.332 |
| E + S+ L | 11 (9.65) | 4 (6.15) | 49 (13.35) | 0.186 |
| **Renal failure** | 18 (15.79) | 7 (10.77) | 41 (11.17) | 0.393 |
| **Infectious disease** | 34 (29.82) | 33 (50.77) | 155 (42.23) | 0.013 |

**Table S11-1. Statistical results for frequency of digestive organs, renal failure, and infectious disease in each subtype.**

The cancer type classification is based on ICD10 codes. The values in the table are presented in the format of number (percentage), where ** indicates α < .01. The p-values in the right column represent the results of the chi-square test.

Reference

[1] Evans, W. J., Morley, J. E., Argilés, J., Bales, C., Baracos, V., Guttridge, D., Jatoi, A., Kalantar-Zadeh, K., Lochs, H., Mantovani, F., Marks, D., Mitch, W.E., Muscaritoli, M., Najand, A., Ponikowski, P., Fanelli, F.R., Schambelan, M., Schols, A., Schuster, M., Thomas, D., Wolfe, R., Anker, S.D.(2008). Cachexia: a new definition. Clin. Nutr. 27, 793–9. <https://doi.org/10.1016/j.clnu.2008.06.013>.

[2] Fukuura, T. (2011). Cancer cachexia and eicosatetraenoic acid as inflammation in colorectal cancer patients. Nutrition – Japan. Jour. of Nutr. Assess., 28(3), 201-204.
