# Supplementary material for: Predicting mortality dynamics in cancer patients: A machine learning approach to pre-death events
Source: PLoS One. 2025 Sep 9;20(9):e0331650. doi: 10.1371/journal.pone.0331650 (PMC12419616; doi:10.1371/journal.pone.0331650)
Supplement: S1 Text — S1 File. Supplemental information of methodology. S2 File. Laboratory parameter list. S3 File. Performances and confusion matrices of continuous mortality prediction models. S4 File. Mean SHAP values of all parameters immediately before death. S5 File. Reference values of ALB, CRP, BUN, and LDH. S6 File. Details of visualizing changes in patient states using time-series SHAP values. S7 File. Evaluation of the number of clusters in patient stratification using SHAP values. S8 File. Stratification of patient states using laboratory values. S9 File. SHAP behaviors of the top influential items for each subtype. S10 File. Statistical tests on laboratory test values, biological sex, age, and cancer type. S11 File. Detailed analysis and discussion of the background of the patient state change subtypes. (ZIP) [file pone.0331650.s001.zip › supplemental_data_20250407/supplemental_data_s2.docx]

**Supplemental Data S2 Laboratory parameter list**

As a result of data selection (See Methods and Fig 1 of S1 Appendix), 77 laboratory parameters were included in the dataset. These features are shown in Table S2-1, including 53 blood test items and 24 urine test items.


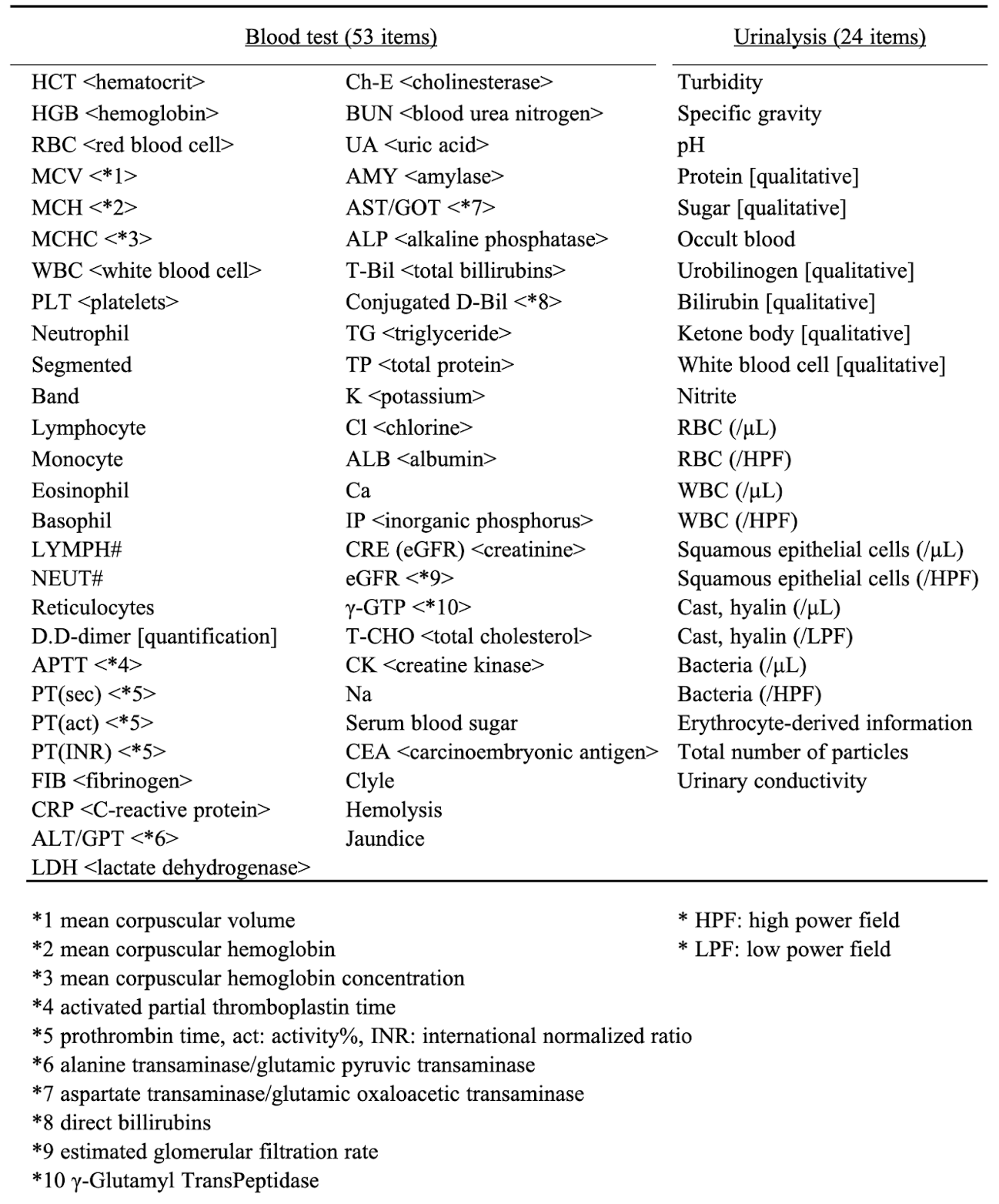


**Table S2-1. Laboratory parameters in the time-series EHR data for analysis.**

The 77 laboratory test items selected through data preprocessing. Items marked with [qualitative] indicate that they are qualitative tests. <test item name> indicates the full name of the test item.
