# Supplementary material for: Predicting mortality dynamics in cancer patients: A machine learning approach to pre-death events
Source: PLoS One. 2025 Sep 9;20(9):e0331650. doi: 10.1371/journal.pone.0331650 (PMC12419616; doi:10.1371/journal.pone.0331650)
Supplement: S1 Text — S1 File. Supplemental information of methodology. S2 File. Laboratory parameter list. S3 File. Performances and confusion matrices of continuous mortality prediction models. S4 File. Mean SHAP values of all parameters immediately before death. S5 File. Reference values of ALB, CRP, BUN, and LDH. S6 File. Details of visualizing changes in patient states using time-series SHAP values. S7 File. Evaluation of the number of clusters in patient stratification using SHAP values. S8 File. Stratification of patient states using laboratory values. S9 File. SHAP behaviors of the top influential items for each subtype. S10 File. Statistical tests on laboratory test values, biological sex, age, and cancer type. S11 File. Detailed analysis and discussion of the background of the patient state change subtypes. (ZIP) [file pone.0331650.s001.zip › supplemental_data_20250407/supplemental_data_s3.docx]

**Supplemental Data S3 Performances and confusion matrices of continuous mortality-prediction models**

Figure S3-1 shows the performance of the mortality prediction model at each time point. The performances are calculated as the mean AUROC of the five-fold cross-validation. The closer to the time of death, the higher the performance.


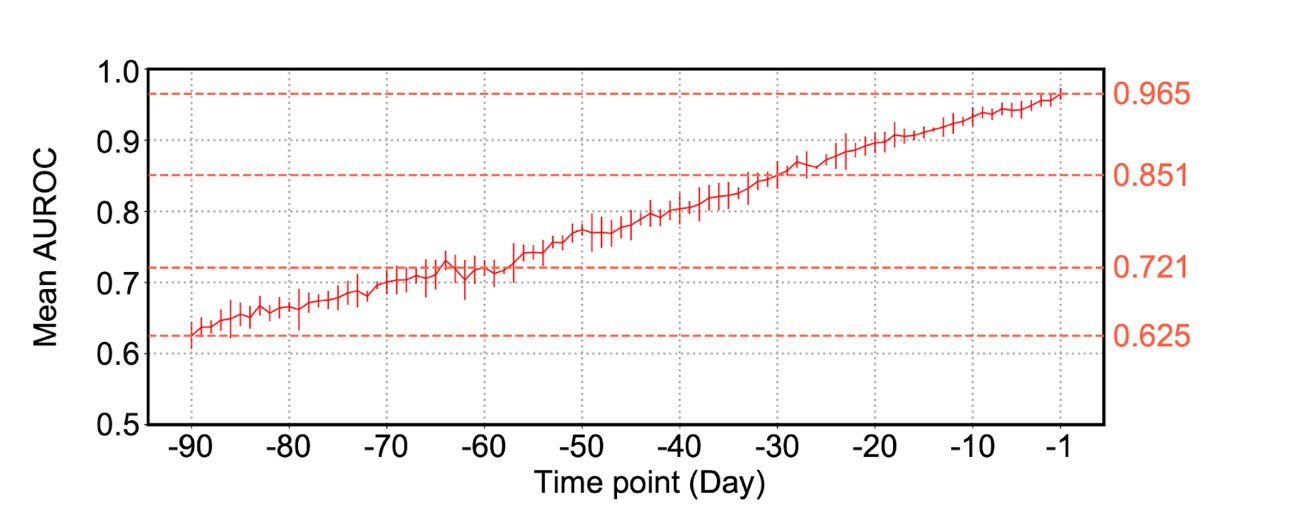


**Fig S3-1. Performance of continuous mortality-prediction models.**

The performances of the continuous mortality-prediction models. The horizontal axis represents the number of days before death. The vertical axis indicates the mean AUROC from five-fold cross-validation. The values of generalization performances at one day, 30 days, 60 days, and 90 days before death are annotated on the right side of the figure. The error bars at each time point represent the range of mean value ±1 SE.

Table S3-1 shows the confusion matrices of the mortality-prediction models every 10 days from 10 days to 90 days before death, and one day before death.

| **Time point (Day)** | **TP** | **FN** | **FP** | **TN** |
| --- | --- | --- | --- | --- |
| -1 | 736 | 72 | 59 | 723 |
| -10 | 847 | 154 | 96 | 789 |
| -20 | 916 | 233 | 136 | 819 |
| -30 | 899 | 297 | 169 | 771 |
| -40 | 934 | 370 | 196 | 760 |
| -50 | 975 | 510 | 149 | 614 |
| -60 | 934 | 570 | 174 | 538 |
| -70 | 1051 | 695 | 173 | 529 |
| -80 | 589 | 443 | 562 | 708 |
| -90 | 882 | 768 | 313 | 427 |

**Table S3-1. Confusion matrices at every 10 time points.**

The confusion matrix at each prediction time point. The abbreviation stands for the following: TP (True Positive), FN (False Negative), FP (False Positive), and TN (True Negative). These are represented as the sum of the confusion matrices of the five models in cross-validation. The sum of TP and FN, as well as the sum of TN and FP, remains the same at each time point (detailed in S1 Appendix), matching the number of patient samples at each time point shown in Fig 3A of S1 Appendix.
