# Supplementary material for: Predicting mortality dynamics in cancer patients: A machine learning approach to pre-death events
Source: PLoS One. 2025 Sep 9;20(9):e0331650. doi: 10.1371/journal.pone.0331650 (PMC12419616; doi:10.1371/journal.pone.0331650)
Supplement: S1 Text — S1 File. Supplemental information of methodology. S2 File. Laboratory parameter list. S3 File. Performances and confusion matrices of continuous mortality prediction models. S4 File. Mean SHAP values of all parameters immediately before death. S5 File. Reference values of ALB, CRP, BUN, and LDH. S6 File. Details of visualizing changes in patient states using time-series SHAP values. S7 File. Evaluation of the number of clusters in patient stratification using SHAP values. S8 File. Stratification of patient states using laboratory values. S9 File. SHAP behaviors of the top influential items for each subtype. S10 File. Statistical tests on laboratory test values, biological sex, age, and cancer type. S11 File. Detailed analysis and discussion of the background of the patient state change subtypes. (ZIP) [file pone.0331650.s001.zip › supplemental_data_20250407/supplemental_data_s8.docx]

**Supplemental Data S8 Stratification of patient states using laboratory values**

For comparison with the stratification of patient states using SHAP values, we clustered patient states based on laboratory test values. Test values were standardized before clustering. As a result, four subtypes were obtained (Fig S8-1A). The number of subtypes was assessed using the silhouette score and ELBOW method (Fig S8-1B, C).

Subsequently, we visualized the SHAP behavior over the 90 days preceding death for each trajectory to patient state subtypes (Fig S8-1D). While the importance of ALB was higher in trajectory 1* and trajectory 3*, the importance of LDH was higher in trajectory 4*, showing slight differences between subtypes. However, these tendencies were also captured in the subtypes based on SHAP values (Fig 5). In the SHAP-based subtypes, we were able to capture crucial characteristics, such as the extremely small influence of ALB in trajectory 1 and the CRP in trajectory 2. These results suggest that the clustering of patient states using SHAP values (Fig 4) is superior to clustering based on laboratory test values in classifying the transitions of patient states leading to death.


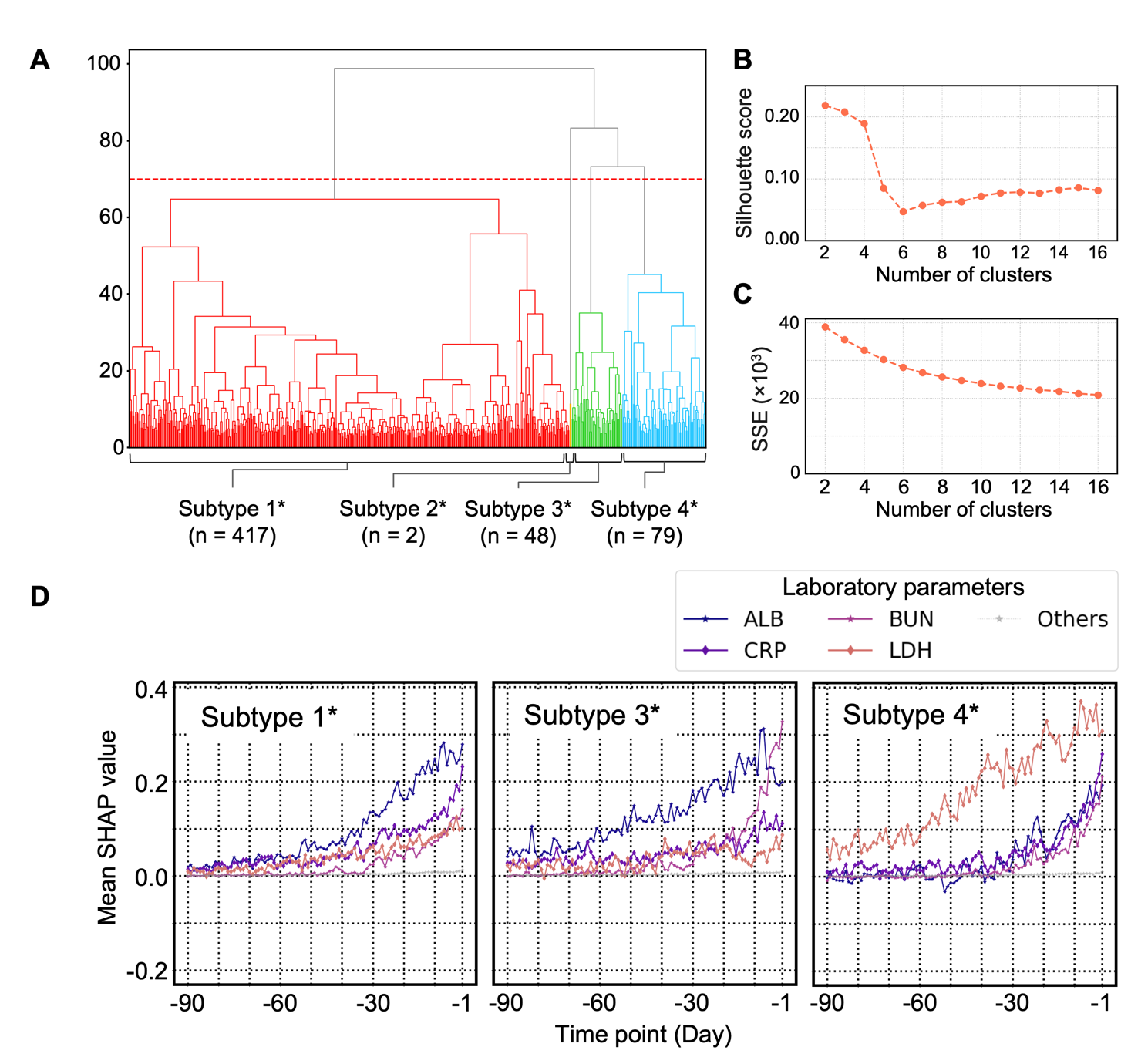


**Fig S8-1. Classification of patient state subtypes using laboratory test values.**

**(A)** Stratification of patient state subtypes on one day before death. The horizontal axis represents each patient sample on the day before death. Clustering was performed using Ward's method and Euclidean distance. The red dashed line indicates the threshold. The number of patient samples in each subtype is shown at the bottom of the plot. To distinguish from the stratification results based on SHAP values, subtype names are denoted with an asterisk (*). **(B)** Evaluation of the number of clusters using the silhouette score. The horizontal axis represents the number of clusters, and the vertical axis represents the silhouette coefficient. **(C)** Evaluation of the number of clusters using the ELBOW method. The vertical axis represents SSE. **(D)** SHAP behavior for each trajectory leading to patient state subtypes. Subtype 2* was excluded due to insufficient sample size. Although variations in highly influential features were captured to some extent, unlike the SHAP-based case, changes in minimal important features were not captured.
