# Supplementary material for: Predicting mortality dynamics in cancer patients: A machine learning approach to pre-death events
Source: PLoS One. 2025 Sep 9;20(9):e0331650. doi: 10.1371/journal.pone.0331650 (PMC12419616; doi:10.1371/journal.pone.0331650)
Supplement: S1 Text — S1 File. Supplemental information of methodology. S2 File. Laboratory parameter list. S3 File. Performances and confusion matrices of continuous mortality prediction models. S4 File. Mean SHAP values of all parameters immediately before death. S5 File. Reference values of ALB, CRP, BUN, and LDH. S6 File. Details of visualizing changes in patient states using time-series SHAP values. S7 File. Evaluation of the number of clusters in patient stratification using SHAP values. S8 File. Stratification of patient states using laboratory values. S9 File. SHAP behaviors of the top influential items for each subtype. S10 File. Statistical tests on laboratory test values, biological sex, age, and cancer type. S11 File. Detailed analysis and discussion of the background of the patient state change subtypes. (ZIP) [file pone.0331650.s001.zip › supplemental_data_20250407/supplemental_data_s4.docx]

**Supplemental Data S4 Mean SHAP values of all parameters immediately before death**

The mean SHAP values for all 77 laboratory parameters are calculated one day before death, including the top 10 influential parameters. The results are shown in Fig S4-1. The top 10 influential parameters accounted for approximately 75% of the SHAP values overall. Among them, ALB, CRP, BUN, and LDH, the top 4 features, had particularly large mean SHAP values.


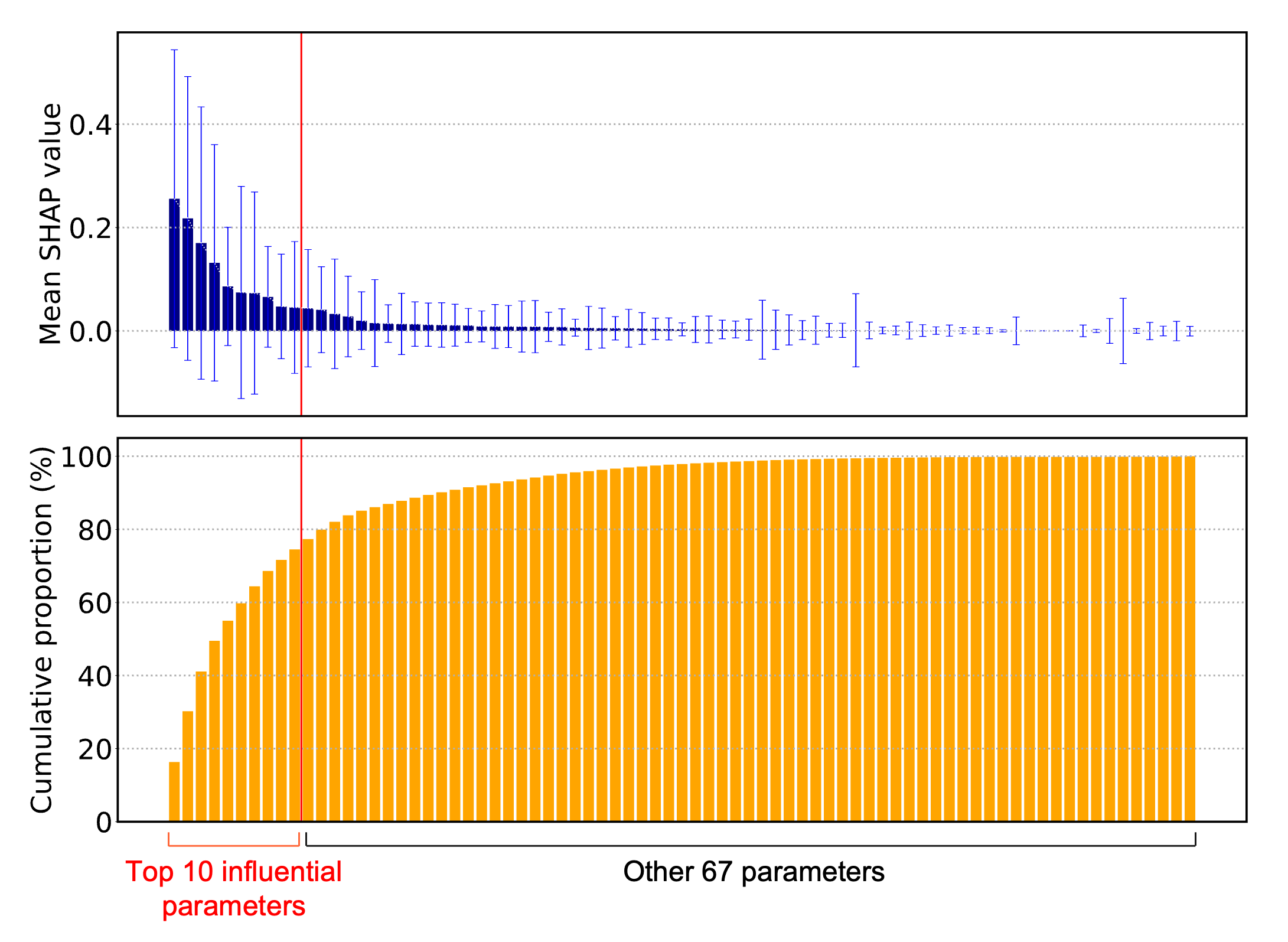


**Fig S4-1. Mean SHAP value of all laboratory parameters one day before death.**

The mean SHAP values for the 77 parameters at one day before death. The vertical axis indicates the mean of SHAP values, normalized by the maximum value in the SHAP value dataset. A red line is drawn between the top influential parameters (top 10 features) and the rest. (Top) Mean SHAP values for each parameter. Error bars represent the range of mean ± 1SE. (Bottom) Cumulative percentage of the absolute mean SHAP values against the total sum of absolute mean SHAP values. The top 10 influential parameters constitute approximately 75% of the total.
