# Supplementary material for: Predicting mortality dynamics in cancer patients: A machine learning approach to pre-death events
Source: PLoS One. 2025 Sep 9;20(9):e0331650. doi: 10.1371/journal.pone.0331650 (PMC12419616; doi:10.1371/journal.pone.0331650)
Supplement: S1 Text — S1 File. Supplemental information of methodology. S2 File. Laboratory parameter list. S3 File. Performances and confusion matrices of continuous mortality prediction models. S4 File. Mean SHAP values of all parameters immediately before death. S5 File. Reference values of ALB, CRP, BUN, and LDH. S6 File. Details of visualizing changes in patient states using time-series SHAP values. S7 File. Evaluation of the number of clusters in patient stratification using SHAP values. S8 File. Stratification of patient states using laboratory values. S9 File. SHAP behaviors of the top influential items for each subtype. S10 File. Statistical tests on laboratory test values, biological sex, age, and cancer type. S11 File. Detailed analysis and discussion of the background of the patient state change subtypes. (ZIP) [file pone.0331650.s001.zip › supplemental_data_20250407/supplemental_data_s5.docx]

**Supplemental Data S5 Reference values of ALB, CRP, BUN, and LDH**

The reference values for ALB, CRP, BUN, and LDH are shown in Table S5-1. The reference values were based on the Department of Clinical Laboratory, Kyoto University Hospital [1].


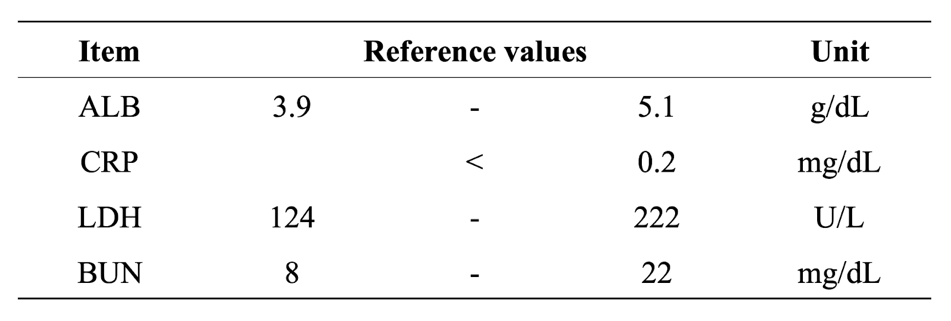


**Table S5-1. Laboratory reference values for ALB, CRP, BUN, and LDH.**

Reference

[1] Department of Clinical Laboratory, Kyoto University Hospital. (n.d.). List of reference values for laboratory tests (15th ed.).

<https://clinical-lab.kuhp.kyoto-u.ac.jp/reference/item/appendix_public/pm_common_0005_ver015.pdf>
