# Supplementary material for: Predicting mortality dynamics in cancer patients: A machine learning approach to pre-death events
Source: PLoS One. 2025 Sep 9;20(9):e0331650. doi: 10.1371/journal.pone.0331650 (PMC12419616; doi:10.1371/journal.pone.0331650)
Supplement: S1 Text — S1 File. Supplemental information of methodology. S2 File. Laboratory parameter list. S3 File. Performances and confusion matrices of continuous mortality prediction models. S4 File. Mean SHAP values of all parameters immediately before death. S5 File. Reference values of ALB, CRP, BUN, and LDH. S6 File. Details of visualizing changes in patient states using time-series SHAP values. S7 File. Evaluation of the number of clusters in patient stratification using SHAP values. S8 File. Stratification of patient states using laboratory values. S9 File. SHAP behaviors of the top influential items for each subtype. S10 File. Statistical tests on laboratory test values, biological sex, age, and cancer type. S11 File. Detailed analysis and discussion of the background of the patient state change subtypes. (ZIP) [file pone.0331650.s001.zip › supplemental_data_20250407/supplemental_data_s9.docx]

**Supplemental Data S9 SHAP behaviors of the top influential items for each subtype**

The SHAP behavior of the top 10 influential features for each trajectory leading to patient state subtypes were calculated and visualized (Fig S9-1).


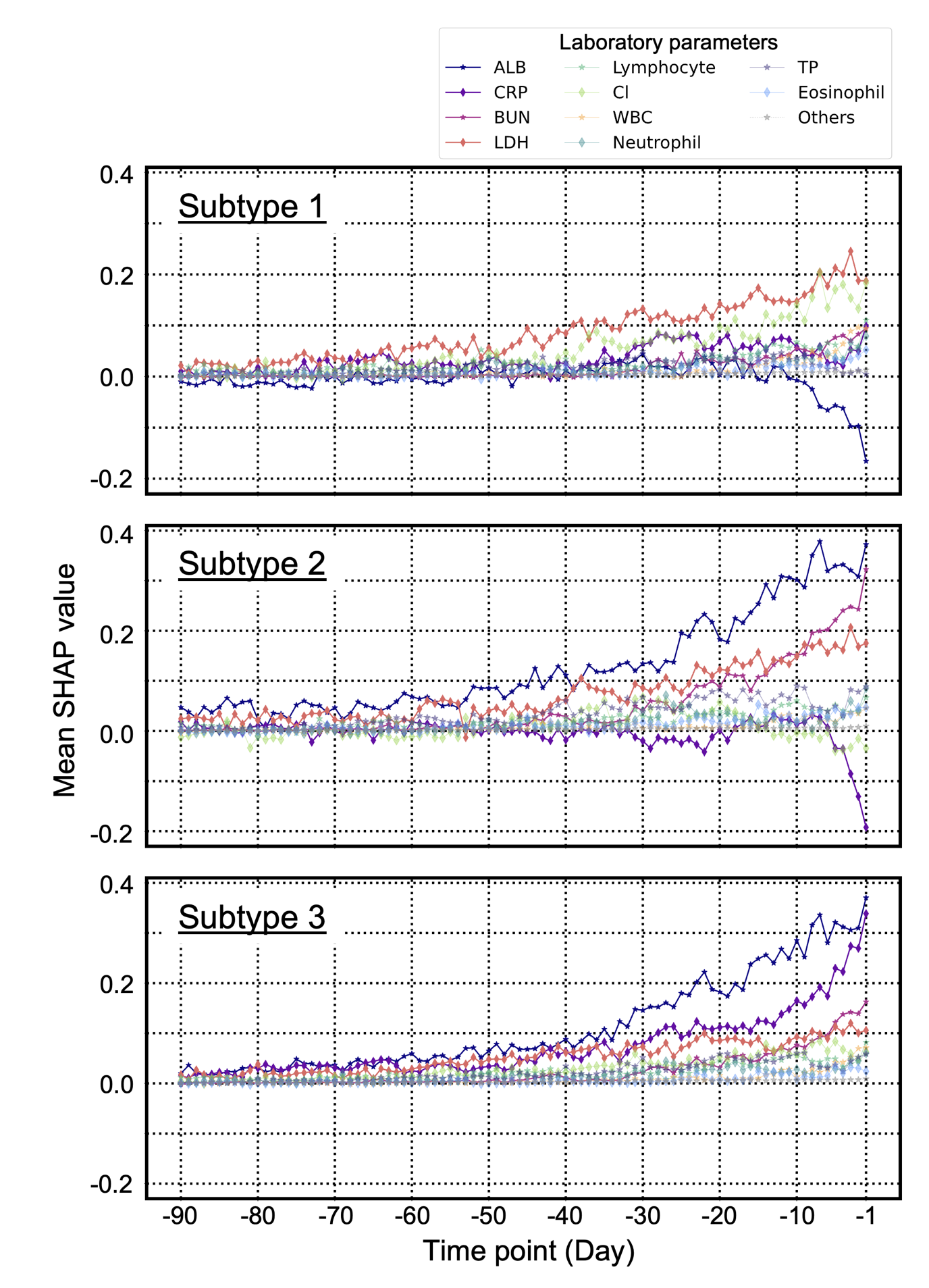


**Fig S9-1**. **SHAP behavior for the top 10 influential features in each patient state subtype.**

The SHAP behavior for the top 10 influential features in each patient state subtype. The vertical axis represents the mean SHAP values scaled by the maximum SHAP value. "Others" represents the mean transition of the SHAP behavior for the 67 parameters excluding the top 10 parameters. The horizontal axis represents the number of days before death. Note that the changes are discrete on a daily basis.
